# Advancing CAR T-Cell Therapy in Solid Tumors: Current Landscape and Future Directions

**DOI:** 10.3390/cancers17172898

**Published:** 2025-09-03

**Authors:** Saeed Rafii, Deborah Mukherji, Ashok Sebastian Komaranchath, Charbel Khalil, Faryal Iqbal, Siddig Ibrahim Abdelwahab, Amin Abyad, Ahmad Y. Abuhelwa, Lakshmikanth Gandikota, Humaid O. Al-Shamsi

**Affiliations:** 1 Department of Oncology, Mediclinic City Hospital, Dubai P.O. Box 505004, United Arab Emirates; 2 Emirates Oncology Society, Dubai P.O. Box 6600, United Arab Emirates; humaid.al-shamsi@medportal.ca; 3 Gulf Cancer Society, P.O. Box 26733, Alsafa 13128, Kuwait; 4 Department of Oncology, Clemenceau Medical Center, Dubai 112963, United Arab Emirates; deborah.mukherji@cmcdubai.ae; 5 Department of Oncology, Burjeel Hospital Oman, 3521 Way, Muscat 112-1465, Oman; ashok.sebastian@burjeel.com; 6 Burjeel Cancer Institute, Burjeel Medical City, Abu Dhabi P.O. Box 92510, United Arab Emirates; charbel.khalil@burjeelmedicalcity.com (C.K.); faryal.iqbal@burjeelmedicalcity.com (F.I.); amin.abyad@burjeelmedicalcity.com (A.A.); 7 Health Research Centre, Jazan University, Jazan 45142, Saudi Arabia; sadiqa@jazanu.edu.sa; 8 Department of Pharmacy Practice and Pharmacotherapeutics, University of Sharjah, Sharjah 27272, United Arab Emirates; ahmad.abuhelwa@sharjah.ac.ae; 9 Immuneel Therapeutics Pvt. Ltd., 8th Floor, Mazumdar Shaw Medical Center, Narayana Hrudayalaya Health City, Hosur Rd, Bommasandra Industrial Area, Bommasandra, Bengaluru 560099, India; lakshmikanth.gandikota@immuneel.com; 10 Department of Medical Oncology, Dana-Farber Cancer Institute, Harvard Medical School, Boston, MA 02215, USA; 11 Harvard Medical School, Harvard University, Boston, MA 20138, USA; 12 College of Medicine, Ras Al Khaimah Medical and Health Sciences University, Al Juwais, Al Qusaidat, Ras Al Khaimah 11172, United Arab Emirates; 13 College of Medicine, Gulf Medical University, Ajman P.O. Box 4184, United Arab Emirates; 14 College of Medicine, University of Sharjah, Sharjah P.O. Box 27272, United Arab Emirates

**Keywords:** CAR T, allogenic CAR T, cellular therapy, solid tumors, gene-edited T cells, T-cell receptor (TCR) therapy

## Abstract

Background: Chimeric Antigen Receptor (CAR) T-cell therapy has transformed the treatment of hematological malignancies, yet its application in solid tumors remains constrained by unique biological and logistical barriers. Objective: This review critically examines the evolving landscape of CAR T-cell therapy in solid malignancies, with a focus on antigen heterogeneity, the immunosuppressive tumor microenvironment, and risks of on-target, off-tumor toxicity. Methods: We outline recent advances in CAR engineering, including co-stimulatory optimization, dual- and multi-antigen targeting, armored CARs, and gene-edited constructs designed to enhance persistence and anti-tumor activity. Clinical progress is highlighted by recent FDA approvals of genetically modified T-cell therapies in synovial sarcoma and melanoma, underscoring the potential for broader solid tumor application. Additionally, we synthesize early-phase clinical trial findings across multiple solid tumor types (e.g., lung, colorectal, ovarian, glioblastoma), and discuss innovative approaches such as regional delivery, checkpoint blockade combinations, and incorporation of chemokine receptors for improved tumor infiltration. The review also considers future strategies, including artificial intelligence-guided target discovery and rational trial design to overcome translational bottlenecks. Conclusions: With expanding clinical experience and continued technological innovation, CAR T-cell therapy is steadily transitioning from an experimental strategy to a therapeutic reality in solid tumors, poised to reshape the future of cancer immunotherapy.

## Simple Summary

Cell therapy has shown remarkable success in treating certain blood cancers, but using it to treat solid tumors like lung, colon, or brain cancer is still a major challenge. These types of cancers are harder to target because their cells vary a lot and create an environment that weakens immune responses. In this review, we explore the current state of CAR T-cell therapy in solid tumors, reasons for past limitations, and how new scientific advances may help overcome them. We discuss exciting progress such as better design of CAR T-cells, new ways to deliver them to tumors, and combining them with other treatments. We also highlight promising results from early clinical trials and look ahead to how technologies like artificial intelligence could help guide future research. This work aims to provide researchers and clinicians with an up-to-date overview and help guide the next steps in making CAR T-cell therapy more effective for solid tumors.

## 1. Introduction

Cancer immunotherapy has emerged as a paradigm-shifting approach in cancer treatment, harnessing the body’s immune system to recognize and eliminate malignant cells. Among these strategies, chimeric antigen receptor (CAR) T-cell therapy represents one of the most significant breakthroughs, particularly in hematological malignancies. The remarkable success of CAR T-cells in treating relapsed/refractory B-cell malignancies has led to several U.S. Food and Drug Administration (FDA) approvals, including tisagenlecleucel (Kymriah) and axicabtagene ciloleucel (Yescarta), transforming treatment outcomes for patients with these previously difficult-to-treat conditions [1,2,3]. While success in hematological malignancies has been striking, with complete remission rates exceeding 80% in some B-cell malignancies [4], extending this therapeutic modality to solid tumors has proven considerably more challenging. Solid tumors’ microenvironment presents a set of formidable barriers, including heterogeneous antigen expression, an immunosuppressive microenvironment, and physical obstacles to T-cell infiltration and function [5,6]. Overcoming these obstacles is crucial to expanding the therapeutic potential of CAR T-cell therapy to a wider range of cancers [7].

The potential impact of successful CAR T-cell therapy for solid tumors, which constitute approximately 90% of all human cancers, cannot be overstated. The 2024 FDA approval of T-cell receptor (TCR) therapy afamitresgene autoleucel for advanced synovial sarcoma, while not a CAR T product, marks genetically modified T-cell therapy approved for a solid tumor and underscores the feasibility of cell-based immunotherapy in this setting.

This review comprehensively examines the current landscape of CAR T-cell therapy in solid tumors, analyzing both the successes and limitations observed in clinical trials. We explore the fundamental challenges unique to solid tumors, highlight innovative strategies being developed to overcome these barriers, and discuss the most promising applications in specific tumor types. Finally, we present emerging technologies and future directions that may expand the therapeutic reach of CAR T-cells in solid malignancies.

## 2. Principles of CAR T-Cell Therapy

CAR T-cell therapy is a form of adoptive cellular immunotherapy in which patient- or donor-derived T lymphocytes are genetically modified to express synthetic receptors, CARs, that specifically target tumor-associated antigens (TAAs). These CARs combine the antigen-binding domains of monoclonal antibodies with intracellular T-cell signaling domains to induce T-cell activation independently of Major Histocompatibility Complex (MHC) recognition.

CARs are typically structured with the following: (1) an extracellular single-chain variable fragment (scFv) derived from a monoclonal antibody; (2) a hinge/spacer and transmembrane domain; and (3) intracellular signaling motifs such as CD3ζ (the primary activation signal, (signal 1) and one or more costimulatory domains (e.g., CD28 or 4-1BB) that enhance T-cell proliferation and persistence (signal 2). Generations of CARs have evolved from first-generation (CD3ζ only) to second- and third-generation constructs incorporating one or two costimulatory domains, respectively, and more recently, fourth-generation “armored” CARs that secrete cytokines (e.g., IL-12), or express immune checkpoint modulators [8,9].

**Figure 1:**
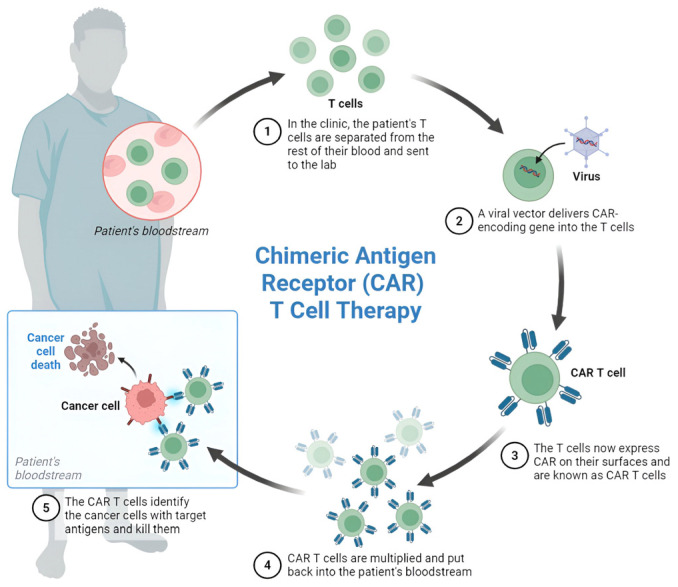
The manufacturing process of CAR T-cells.

The canonical CAR T-cell manufacturing workflow includes the following key steps illustrated in Figure 1:

1. Leukapheresis: Collection of the patient’s peripheral blood mononuclear cells.
2. Ex vivo T-cell isolation and activation: Selection and stimulation of T-cells.
3. Genetic modification: Introduction of the CAR construct through viral vectors (typically retrovirus or lentivirus) or non-viral methods.
4. Cell expansion: Cultivation of modified T-cells to achieve therapeutic quantities.
5. Quality control: Testing for sterility, identity, and potency.
6. Cryopreservation: Freezing for transportation and storage.
7. Lymphodepletion conditioning: Pre-treatment conditioning of the patient (e.g., cyclophosphamide/fludarabine).
8. Infusion: Administration of the CAR T-cell product [10,11,12,13].

These steps form the foundation for generating functional CAR T-cells, which are then characterized by their structural components and functional mechanisms, as described further.

## 3. CAR T-Cell Construction

The construction of CAR T-cells involves the genetic engineering of T-cells to express a CAR capable of recognizing specific TAAs. While the basic CAR structure includes an extracellular recognition domain, a transmembrane region, and intracellular signaling modules, the design of each component can be fine-tuned to optimize antigen specificity, stability, and T-cell function.

1. Extracellular Antigen Recognition Domain: The recognition domain is derived from a monoclonal antibody and is responsible for targeting specific antigens on the surface of tumor cells. Commonly, single-chain variable fragments (scFvs) are utilized due to their ability to combine the specificity of antibodies with the advantages of smaller size and increased stability. Although traditionally derived from murine antibodies, efforts to humanize scFvs are underway to reduce immunogenicity and enhance in vivo persistence.
2. Transmembrane Domain: This domain anchors the CAR molecule to the T-cell membrane, thereby enabling the transduction of signals upon antigen engagement. It plays a crucial role in maintaining the structural integrity and functionality of the CAR.

In addition to structural support, the hinge domain regulates the CAR signaling threshold, while the transmembrane domain modulates the magnitude of CAR signaling by controlling receptor expression levels [14].

3. Intracellular Signaling Domains: The intracellular domain includes a combination of CD3ζ and co-stimulatory domains such as CD28, 4-1BB (CD137), or OX40 (CD134). These signaling domains are pivotal for T-cell activation, proliferation, and survival post-target recognition [15].

The most common method for CAR construction involves the use of viral vectors, such as lentiviruses or retroviruses, which introduce the CAR transgene into the T-cell genome. After transduction, the T-cells are expanded ex vivo in the presence of cytokines and other growth factors (e.g., IL-2, IL-15), prior to infusion back into the patient. Alternatively, non-viral approaches, such as transposon- or CRISPR/Cas9-mediated gene editing, are also being explored to enhance the efficiency and safety of CAR T-cell generation.

Antibody-coated magnetic beads, such as anti-CD3/CD28 Dynabeads, are widely used in CAR T-cell manufacturing to activate and expand T-cells ex vivo, supporting efficient proliferation and scalable production [16].

## 4. Mechanism of Action of CAR T-Cells

CAR T-cells primarily operate through a multi-faceted approach involving direct cytotoxicity, immune signaling, and the modulation of the tumor microenvironment (TME). Upon reinfusion, CAR T-cells recognize tumor cells through their engineered surface receptors, which bind to specific TAAs expressed on the surface of cancer cells. This receptor-mediated binding triggers a series of intracellular signaling cascades that activate T-cell functions, Table 1.

The first critical event following the recognition of the target cell is the formation of the immunological synapse, which establishes close contact between the CAR T-cell and the tumor cell. This interaction induces T-cell activation through the engagement of co-stimulatory domains present in CAR constructs, which are essential for robust T-cell expansion and sustained functionality [17]. The primary cytotoxic effects of activated CAR-T-cells are mediated by the release of granule proteins, such as perforin and granzymes, which induce apoptosis in target cancer cells. Additionally, CAR T-cells can produce pro-inflammatory cytokines, including tumor necrosis factor-alpha (TNF-α) and interferon-gamma (IFN-γ), which facilitate further recruitment and activation of other immune-effector cells [18].

Moreover, CAR T-cells can also exert indirect anti-tumor effects by modulating the immune microenvironment. The release of these cytokines and chemokines can enhance immune infiltration within the tumor, overcoming the immunosuppressive factors often present in solid tumors [19].

## 5. Evolution of CAR T-Cell Therapy

Since its inception, CAR T-cell therapy has evolved significantly, giving rise to multiple generations of CAR constructs with distinct design features aimed at enhancing therapeutic efficacy, persistence, and safety.

First-Generation CARs: The initial constructs consisted solely of the CD3ζ signaling domain and an extracellular scFv for tumor recognition, lacking co-stimulatory signals required for sustained activation and proliferation. While first-generation CAR T-cells demonstrated proof of concept, showing activity in vitro and some clinical responses, they often exhibited limited efficacy, suboptimal expansion in vivo, and a tendency to undergo rapid exhaustion without sufficient co-stimulation [20]. These limitations prompted the development of more sophisticated CAR constructs that incorporated additional signaling domains.

Second-Generation CARs: The introduction of co-stimulatory domains such as CD28 or 4-1BB marked the advancement to second-generation CARs. These co-stimulatory motifs facilitate enhanced T-cell activation, proliferation, and persistence following antigen engagement. Clinical trials have demonstrated that second-generation CAR T-cells can elicit stronger anti-tumor responses and improved durability compared to their first-generation counterparts. A notable example is the CD19-targeting CAR T-cells, which have shown remarkable efficacy in treating B-ALL and some lymphomas [21].

Third-Generation CARs: The evolution continued with the advent of third-generation CARs, which integrate multiple co-stimulatory domains (e.g., CD28 and 4-1BB or CD137) along with the CD3ζ signaling domain. This intricate design aims to amplify T-cell activation and enhance their survival and functionality within the tumoral environment. Early preclinical studies have suggested that third-generation CARs can offer improved anti-tumor activity and sustained persistence post-administration compared to previous generations [22].

Fourth-Generation CARs: Also known as “TRUCKs” (T-cell Redirected for Universal Cytokine Killing), they are engineered to include a transgenic cytokine payload. These CAR T-cells not only target specific tumor antigens but also secrete cytokines, such as IL-12 or IL-18, to enhance T-cell activation and recruit innate and adaptive immune cells to the tumor site. This “armored” feature helps overcome the immunosuppressive TME and enhances tumor destruction. Preclinical studies and early clinical trials have shown promising results in solid tumors and hematologic malignancies, demonstrating improved anti-tumor efficacy. However, these approaches still face challenges, including uncontrolled cytokine secretion, off-tumor toxicity, and limited persistence in the TME [10].

Fifth-Generation CARs and Beyond: Fifth generation “Armored” CAR T-cells represent a significant advancement in CAR T-cell therapy, incorporating dual-targeting, cytokine secretion, and synthetic Notch (synNotch) receptors to overcome the limitations of previous generations. These CAR T-cells are engineered to target two antigens, reducing antigen escape and improving specificity, while cytokine secretion (e.g., IL-12, IL-15) enhances T-cell activation and overcomes the immunosuppressive TME [23]. The addition of synNotch receptors enables a more selective activation process, ensuring CAR T-cells are only triggered when two distinct tumor markers are present. The fifth-generation CAR T-cells hold significant potential for treating both hematologic and solid tumors and preclinical studies have shown promising results in various cancer models, including glioblastoma, melanoma, and breast cancer, demonstrating enhanced anti-tumor efficacy and reduced toxicity [24,25,26].

The generation of CARs continues to evolve, reflecting advances in immunology, genomics, and synthetic biology. Each successive generation has sought to overcome the limitations of the previous ones, progressively improving and enhancing the therapeutic potential, safety profile, and translational applicability of CAR T-cell therapy [20].

Once autologous CAR T-cell became a proven therapeutic option, the possibility of engineering CAR T-cells from a healthy donor rather than a patient’s own cells was investigated. This key difference enables allogeneic therapies to be manufactured in advance, creating an “off-the-shelf” product that can be readily available for immediate treatment, addressing the long manufacturing time and variability associated with autologous CAR T. The main advantages of allogeneic CAR T include scalability, capacity for repeated dosing, reduced production time, and lower costs. However, the use of donor cells in allogeneic CAR T carries the risk of immune rejection and GVHD, often necessitating immunosuppressive therapy to prevent these complications, which may in turn increase infection risk. Clinical studies have noted potentially higher remission rates and more durable CAR T-cell persistence with allogeneic products, especially when donor and recipient are closely matched, but also highlight the need to balance toxicity and immune compatibility [27]. Although allogenic CARs share some of the toxicities of autologous CARs like CRS, ICANS and prolonged cytopenias, they occur to a lesser degree and are more manageable. However, the potential of GvHD and allogenic CAR rejection by the host T-cells and NK cells are of more concern. The rare yet worrisome potential for insertional mutagenesis, which can cause secondary malignancies, is being studied at present. The current focus of research has been the utilization of gene editing techniques such as CRISPR-Cas9, Zinc finger nucleases (ZFNs), and transcription activator-like effector nucleases (TALENs) to remove endogenous T-cell receptors and other immunogenic markers. Despite current safety and efficacy challenges, ongoing advances in genome editing and immune evasion strategies suggest that allogeneic CAR T-therapies have strong potential to become more widely accessible and clinically competitive with, or even superior to, autologous CAR T-treatments.

## 6. Approved CAR T-Cell Therapies in Hematological Cancers

CAR T-cell therapy has transformed the treatment landscape for various hematological malignancies. Multiple CAR T-cell products have gained approval from the FDA, highlighting the ever-growing number of hematological cancers that are being treated using CAR T-cell therapy.

### 6.1. Kymriah (Tisagenlecleucel)

Indication: Kymriah was the first CAR T-cell therapy approved by the FDA in August 2017 for the treatment of young patients (up to age 25) with acute lymphoblastic leukemia (ALL) that is refractory or in second or later relapse [28]. It was also approved for adult patients with large B-cell lymphoma (LBCL) after two or more lines of systemic therapy [29].

Mechanism: The therapeutic efficacy of Kymriah derives from its design to target CD19, a pan-B-cell antigen. This is accomplished through a CAR construct engineered to redirect the patient’s T-cells to recognize and induce apoptosis in CD19-expressing malignant B cells.

### 6.2. Yescarta (Axicabtagene Ciloleucel)

Indication: Yescarta received FDA approval in October 2017 for the treatment of adult patients with refractory LBCL who have not responded to previous treatments (refractory) or who have relapsed after two or more lines of therapy [30].

Mechanism: Similarly to Kymriah, Yescarta is designed to target CD19. It employs a different CAR construct, comprising a CD28 co-stimulatory domain that enhances T-cell expansion and persistence, contributing to its efficacy.

### 6.3. Breyanzi (Lisocabtagene Maraleucel)

Indication: Breyanzi was granted FDA approval in February 2021 for the treatment of adult patients with LBCL who have not responded to two or more prior lines of systemic therapy [31].

Mechanism: Breyanzi, similar to its predecessors, targets CD19 through a novel CAR construct that integrates a 4-1BB co-stimulatory domain. This unique configuration is instrumental in enhancing the durability and functional potential of the modified T-cells.

### 6.4. Abecma (Idecabtagene Vicleucel)

Indication: Abecma was approved in March 2021, marking it as the first CAR T-cell therapy sanctioned for the treatment of multiple myeloma [32]. It is specifically indicated for adult patients who have undergone at least four prior lines of therapy, including treatment with a proteasome inhibitor and an immunomodulatory agent.

Mechanism: Abecma targets BCMA (B-cell maturation antigen), a protein primarily expressed on malignant plasma cells. The CAR structure includes a CD3ζ signaling domain coupled with a co-stimulatory 4-1BB domain, facilitating effective T-cell activation, cytokine production, and cytotoxic activity.

### 6.5. Carvykti (Ciltacabtagene Autoleucel)

Indication: Approved in March 2022, Carvykti is another option for the treatment of adult patients with multiple myeloma who have received at least four prior therapies [33].

Mechanism: Carvykti similarly targets BCMA, but with engineering adaptations designed to enhance the persistence and activity of T-cells against BCMA-expressing myeloma cells, thus providing another effective avenue for treatment in this patient population.

### 6.6. Tecartus (Brexucabtagene Autoleucel)

Indication: Approved in July 2020 for adult patients with relapsed or refractory mantle cell lymphoma (MCL) who have received at least one prior therapy. Later, approval was extended to relapsed/refractory adult acute lymphoblastic leukemia (ALL).

Mechanism: Tecartus targets the CD19 antigen on malignant B cells. The CAR construct includes a CD28 co-stimulatory domain and CD3ζ signaling domain, promoting robust T-cell activation, expansion, and cytotoxicity against CD19-expressing cancer cells [34].

### 6.7. Aucatzyl (Obecabtagene Autoleucel)

Indication: Approved by the FDA in November 2024 for adults with relapsed or refractory B-cell precursor acute lymphoblastic leukemia (B-ALL) [35].

Mechanism: A CD19-directed CAR T-cell therapy with 4-1BB and CD3ζ co-stimulatory domains, enabling T-cells to recognize and kill CD19-expressing malignant B cells.

## 7. Recent Approvals of Engineered T-Cell Therapies for Solid Tumors

**Figure 2:**
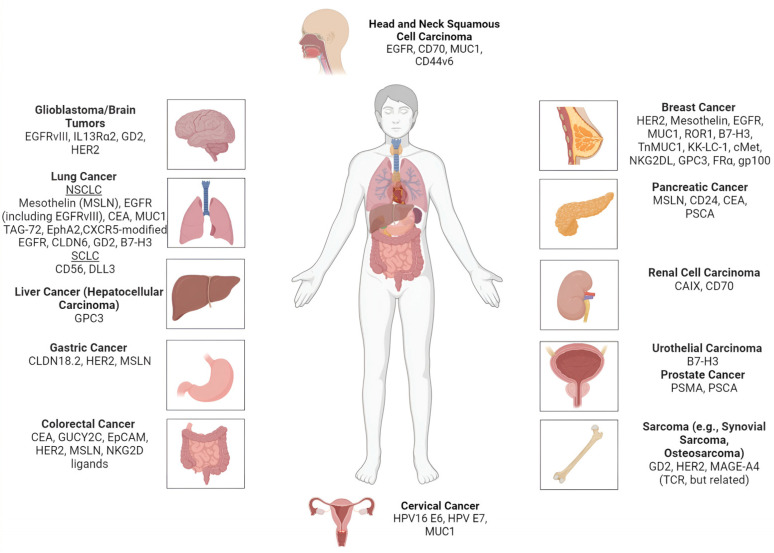
Anatomical Distribution of the major ongoing clinical trials and the potential therapeutic of CAR T-targeted antigens in solid tumors in human. The antigens are specified as follows: Breast Cancer (*HER2, Mesothelin),* Non-Small Cell Lung Cancer (*NSCLC: Mesothelin, EGFR*), Small Cell Lung Cancer (*SCLC: CD56, DLL3*), Colorectal Cancer (*CEA, GUCY2C*), Gastric Cancer (*CLDN18.2, HER2*), Renal Cell Carcinoma (*CAIX, CD70*), Prostate Cancer (*PSMA, PSCA*), Urothelial Carcinoma (*B7-H3*), Cervical Cancer (*HPV16 E6, HPV E7*), Head and Neck Squamous Cell Carcinoma (*EGFR, CD70*), Pancreatic Cancer (*MSLN, CD24*), Liver Cancer (*GPC3*), Sarcoma (*GD2, HER2*), and Glioblastoma (*EGFRvIII, IL13Rα2*). This figure offers a visual representation of the major ongoing clinical trials and the potential therapeutic applications of CAR T-therapy across diverse cancer types, as of August 2025.

CAR T-therapy trials have been expanding across various disease sites in humans, marking a significant evolution in the treatment of solid tumors. This growth is driven by ongoing clinical investigations targeting a diverse array of antigens. Figure 2 shows the anatomical distribution of the major ongoing clinical trials and the potential therapeutic use of CAR T-targeted antigens in solid tumors in humans. And while CAR T-cell therapies have not yet gained widespread approval for solid tumors like they have for hematologic cancers, in 2024, two genetically engineered autologous T-cell therapies, not conventional CAR T-cells, received accelerated FDA approval for solid tumors, marking a milestone in the clinical translation of adoptive cell therapies beyond hematologic cancers. This section discusses recently approved TCR- and TIL-based therapies in solid tumors, which, although distinct from CAR T-cells, share foundational principles of personalized adoptive T-cell therapy.

Melanoma and TILs: Melanoma, an aggressive skin cancer, was the first solid tumor where tumor-infiltrating lymphocytes (TILs) were shown to mediate anti-tumor immunity. Research on TILs, harvesting, expanding, and reinfusing patient-derived immune cells paved the way for CAR T-cell therapies by demonstrating that T-cells can be engineered or selected to effectively target tumors [36].

### 7.1. Synovial Sarcoma

As of April 2025, synovial sarcoma is the only indication that a cell therapy has gained FDA approval for solid cancer. Synovial sarcoma is a rare but aggressive soft tissue sarcoma that typically affects adolescents and young adults.

In the non-randomized Phase II SPEARHEAD-1 trial (NCT04044768), patients with metastatic or unresectable synovial sarcoma or myxoid round cell liposarcoma with MAGE-A4 expression, and who had been previously treated with at least one line of anthracycline-containing or ifosfamide-containing chemotherapy were treated with a single dose of Afamitresgene autoleucel (afami-cel). The investigators reported an objective response rate (ORR) of 36% and disease control rate (DCR) of 71% with median duration of response (DoR) of 17.3 months in 45 patients with synovial sarcoma. Most common AEs were hematologic (neutropenia, lymphopenia), and low-grade cytokine release syndrome (CRS) (22% Grade 1–2); no Grade ≥ 3 CRS or neurotoxicity was observed [37]. Based on these results, afami-cel gained accelerated FDA approval in August 2024 for advanced synovial sarcoma [38]. Although afami-cel is a genetically engineered TCR therapy, and not exactly a conventional CAR T, it represents the first genetically modified T-cell therapy approved in a solid tumor setting, highlighting progress in the broader adoptive cell therapy field.

### 7.2. Malignant Melanoma

Lifileucel, an autologous tumor-infiltrating lymphocyte (TIL) therapy, received accelerated FDA approval in 2024 for the treatment of patients with unresectable or metastatic melanoma who had previously progressed on PD-1 inhibitors and, if applicable, BRAF-targeted therapies [39]. The approval was based on data from the therapy strategies, particularly Checkpoint Inhibitor Trial, a multicenter, single-arm, multicohort Phase II study evaluating the efficacy and safety of lifileucel. Patients received a single infusion of lifileucel following non-myeloablative lymphodepleting chemotherapy, with administered doses ranging from 7.5 × 10^9^ to 72 × 10^9^ viable tumor-infiltrating lymphocytes. Among 73 evaluable patients in Cohort 4 of the study, lifileucel demonstrated an objective response rate (ORR) of 31.5% (95% CI: 21.1–43.4), including both complete and partial responses. Notably, 56.5% of responders maintained their responses for at least six months, and 43.5% achieved a duration of response lasting ≥12 months, demonstrating that durable responses are achievable in select solid tumors through TIL-based therapies [40].

## 8. CAR T-Cell Therapies in Development in Solid Malignancies

While CAR T-cell therapy has revolutionized the treatment of hematologic malignancies, its translation to solid tumors has proven significantly more challenging. The development of CAR T-cell therapies for solid malignancies is a rapidly evolving frontier in oncology, focused on overcoming a unique set of obstacles including the identification of reliable tumor-associated antigens, the immunosuppressive TME, and poor T-cell trafficking and persistence. Current investigative strategies are multifaceted, encompassing next-generation CAR designs with enhanced safety and efficacy profiles, combination approaches to modulate the TME, and novel targets to address tumor heterogeneity. This section will detail the promising CAR T-cell therapies currently in development for solid tumors, highlighting the innovative engineering and clinical strategies being employed to unlock their therapeutic potential.

### 8.1. CAR T-Cell Therapy in Breast Cancer

Breast cancer is the most common cancer in women worldwide, accounting for 1 in 4 female cancers and causing over 669,000 deaths in 2022, making it the leading cause of cancer-related death in women [41].

Despite advances in early detection and targeted therapies, the prognosis for patients with metastatic breast cancer, particularly those with triple-negative breast cancer (TNBC) and HER2-positive breast cancer, remains poor. CAR T-cell therapy has been investigated as a potential therapeutic strategy for breast cancer. This section provides an in-depth review of the ongoing clinical trials and experimental strategies aimed at advancing CAR T-cell strategies for breast cancer, with a focus on antigen targets, clinical trial outcomes, preclinical mechanisms, and immune-related toxicities.

### 8.2. HER2-Targeted CAR T-Cells in HER2-Positive Breast Cancerteraction Activates the T-Cells, Leading to the Release of Cy

HER2 (Human Epidermal Growth Factor Receptor 2) is a receptor tyrosine kinase that is overexpressed in approximately 15–20% of breast cancer cases. HER2-positive breast cancer is characterized by aggressive tumor growth and poor prognosis, but it has benefited from targeted therapies like trastuzumab (Herceptin) and pertuzumab. However, resistance to these therapies often develops, particularly in metastatic disease. CAR T-cell therapy targeting HER2-positive breast cancer has emerged as a promising strategy. The mode of action of HER2-targeted CAR T-cells is based on the ability of the engineered T-cells to recognize and bind specifically to the HER2 antigen on tumor cells. This interaction triggers T-cell activation, cytokine production (e.g., IFN-γ, IL-2), and release of cytotoxic granules (perforin, granzyme B) to mediate tumor cell lysis [42,43].

A phase1/2 trial (NCT00924287) aimed to evaluate the safety and efficacy of HER2-specific CAR T-cells in patients with metastatic HER2-positive breast cancer. However, the trial was terminated following a fatal case of on-target/off-tumor toxicity, in which CAR T-cells attacked low-level HER2-expressing pulmonary epithelial cells resulting in multi-organ failure. These findings underscore the challenges associated with targeting HER2 in solid tumors, particularly concerning off-target effects [44].

One promising strategy includes targeting HER2 variants such as p95HER2 and combining CAR T-cells with bispecific antibodies to enhance activation within the TME [45]. Preclinical studies have demonstrated safety and durable anti-tumor responses in HER2-positive tumor models, supporting ongoing development of clinical trials.

Despite encouraging progress, HER2-targeted CAR T-cell therapy faces challenges, particularly off-target toxicity due to recognition of HER2 expressed on normal tissues, which limits clinical application. To improve therapeutic outcomes, novel CAR T-cell designs such as bispecific CAR T-cells and tandem CAR T-cells (TanCAR T) have emerged. Bispecific CAR T-cells targeting HER2 alongside other tumor-associated antigens (e.g., gp100 or MUC1) have shown enhanced cytotoxicity against breast tumors in preclinical models. TanCAR T-cells incorporate two scFv domains, enabling simultaneous recognition of multiple antigens, which synergistically boosts T-cell activation and anti-tumor effects. For instance, TanCAR T-cells targeting HER2 and other antigens demonstrated improved tumor lysis and cytokine secretion. These multi-targeting CAR T-cell approaches hold significant promise for overcoming limitations of first-generation therapies and improving treatment efficacy for patients with HER2-positive breast cancer [44].

Antigen heterogeneity, such as varying levels of HER2 expression which could lead to the escape of low HER2-expressing tumor cells, remains a challenge. Additionally, the TME in breast cancer, characterized by immune suppression and the presence of myeloid-derived suppressor cells (MDSCs) and regulatory T-cells (Tregs), may hinder CAR T-cell efficacy [43].

### 8.3. Targeting Triple-Negative Breast Cancer (TNBC)

Triple-negative breast cancer (TNBC), characterized by the lack of expression of estrogen receptor (ER), progesterone receptor (PR), and HER2, is often associated with a high risk of metastasis and recurrence, and there are only a few targeted therapies available for this aggressive form of breast cancer. The lack of definitive therapeutic targets in TNBC has led to the exploration of several CAR T-cell strategies targeting other tumor antigens that are expressed in this malignancy.

One of the trials investigating CAR T-cell therapy for TNBC targeted the protein mesothelin, which is overexpressed in many epithelial tumors, including TNBC. The study, a phase 1 trial (NCT05623488), recently terminated due to feasibility concerns, planned to investigate mesothelin-targeted CAR T-cells in patients with advanced TNBC. Mesothelin is an ideal target due to its restricted expression in tumor cells and its role in tumor progression.

Another promising target in TNBC is the epidermal growth factor receptor (EGFR). A phase 1 trial (NCT05341492) is exploring EGFR-targeted CAR T-cells in patients with advanced TNBC. EGFR is a well-known target in several malignancies, including lung cancer, and its overexpression in TNBC provides a potential target for CAR T-cell therapy. Although results could be promising, toxicity remains a significant concern, as EGFR is physiologically expressed in normal epithelial tissues, raising the risk of on-target/off-tumor effects. Table 2 summarizes the completed and ongoing CAR T-cell therapy clinical trials in triple-negative breast cancer.

### 8.4. HER2 CAR T-Cell Therapy and PD-1 Inhibition Combination

Combining HER2-targeted CAR T-cell therapy with immune checkpoint inhibitors, particularly PD-1 blockade, is an emerging strategy to enhance therapeutic efficacy in HER2-positive breast cancer. Preclinical studies have demonstrated that PD-1 signaling can suppress CAR T-cell function within the TME. In one study, third-generation HER2-specific CAR T-cells incorporating CD28 and 4-1BB co-stimulatory domains were evaluated in trastuzumab-resistant breast cancer models. The addition of PD-1 blocking antibodies significantly improved CAR T-cell cytotoxicity and tumor control, suggesting that PD-1 inhibition can restore CAR T-cell activity and overcome resistance mechanisms [46].

### 8.5. Emerging Targets in Breast Cancer

The landscape of CAR T-cell therapy in breast cancer is evolving rapidly, with several emerging targets and strategies under investigation. These include the following:

Glypican-3 (GPC3): A novel target being explored in early-phase clinical trials for its potential to be highly expressed on breast cancer cells while minimally present on normal tissues.

Folate Receptor-Alpha (FRα): Another promising target, especially in patients with refractory breast cancer.

Bispecific CAR T-cells: CAR T-cells engineered to target two antigens simultaneously, potentially overcoming the challenges of tumor heterogeneity [47]. Innovative CAR T-cell designs, such as bispecific and TanCAR T constructs, are emerging as powerful tools in the treatment of HER2-positive breast cancer. Bispecific CAR T-cells, engineered to simultaneously recognize HER2 and a second tumor-associated antigen, such as gp100 or MUC1, have shown the ability to eliminate solid tumors in preclinical models, including those located in the breast and brain of immunocompetent mice. Meanwhile, TanCAR T-cells incorporate two antigen-binding domains within a single receptor, enabling one T-cell to recognize multiple targets. This dual-target engagement can produce a synergistic immune response, improving both cytotoxicity and cytokine secretion. For example, TanCAR T-cells that target both HER2 and CD19 have demonstrated the capacity to destroy tumor cells expressing either antigen while releasing key effector molecules like IFN-γ and IL-2 [48,49]. These advancements highlight the therapeutic promise of multi-targeted CAR T-strategies to address tumor antigen heterogeneity and resistance mechanisms in HER2-driven breast cancers.

Armored CAR T-cells: Armored CAR T-cells are genetically modified to secrete cytokines like IL-12, enhancing their function within the immunosuppressive TME. IL-12 not only boosts CAR T-cell proliferation and cytotoxicity but also reprograms tumor-associated macrophages to support anti-tumor immunity. Preclinical studies show that IL-12-secreting CAR T-cells exhibit improved tumor clearance, increased resistance to checkpoint inhibition, and reduced immune suppression, particularly in models of solid tumors such as ovarian cancer [49].

These strategies are being explored in early-phase trials, and while results are still preliminary, the combination of innovative CAR T-cell designs with advanced immune modulation offers great potential for improving outcomes in breast cancer.

CAR T-cell therapy in breast cancer has shown early promise, particularly in HER2-positive breast cancer, with several clinical trials underway that target different antigens such as mesothelin, EGFR, and folate receptor-alpha. The combination of CAR T-cells with immune checkpoint inhibitors has also demonstrated potential to enhance therapeutic efficacy. However, challenges such as antigen heterogeneity, off-tumor toxicity, and immune evasion remain significant hurdles. Ongoing clinical trials and future innovations in CAR T-cell technology are expected to pave the way for the broader use of this powerful immunotherapy in breast cancer treatment.

## 9. CAR T-Cell Therapy in Lung Cancer

### 9.1. Non-Small Cell Lung Cancer (NSCLC)

Non-small cell lung cancer (NSCLC) remains the leading cause of cancer mortality globally, and while immune checkpoint inhibitors have transformed its management, durable remissions are still achieved in only a minority of patients. Consequently, CAR T-cell therapy is being explored as a strategy to further enhance anti-tumor immunity. However, NSCLC presents considerable challenges for CAR T approaches, including antigen heterogeneity, an immunosuppressive TME, and barriers to T-cell trafficking.

Several TAAs have been targeted in clinical trials of CAR T-therapy in NSCLC. Among the earliest studied was mesothelin, a glycoprotein overexpressed in a subset of NSCLC tumors. Mesothelin (MSLN) is a tumor-associated glycoprotein overexpressed in NSCLC and mesothelioma, associated with poor prognosis and resistance to chemotherapy, and has limited expression in normal tissues, making it an attractive CAR T-cell target. Preclinical studies using second-generation MSLN-targeted CAR T-cells have demonstrated anti-tumor activity but were unable to fully eradicate tumors. Clinically, outcomes have been modest. A Phase I trial (NCT02414269) was terminated due to disease progression and severe toxicities, including one treatment-related death. Ongoing trials continue to evaluate strategies to improve efficacy and safety, including combinations with PD-1 blockade (NCT04577326) and modifications to enhance tumor infiltration, such as CD40L-expressing CAR T-cells (NCT05693844).

EGFR, a receptor tyrosine kinase commonly mutated or overexpressed in NSCLC, has also been targeted using CAR T constructs. While preclinical models using EGFRvIII-targeted CAR T-cells showed efficacy, clinical trials in EGFR-wildtype NSCLC have faced limited efficacy [50,51]. For example, a Phase I trial (NCT01869166) evaluated EGFR-directed CAR T-cells in EGFR-expressing NSCLC. The trial enrolled patients with relapsed or refractory NSCLC after standard therapies and required immunohistochemical confirmation of EGFR expression. Infusions were generally well tolerated, although cytokine release syndrome (CRS) occurred in a minority of patients (all grade 1–2). Some patients exhibited minor tumor reductions, although no complete or partial responses were achieved per RECIST criteria.

A Phase I trial (NCT05060796) using EGFR-targeted CAR T-cells modified with CXCR5 for advanced NSCLC showed manageable toxicity, with one case of grade 3–4 hyperlipasemia. Another trial (NCT04153799) investigating dose optimization reported mild epithelial toxicities in several patients and one case of pulmonary edema.

The tumor-associated glycoprotein-72 (TAG-72) and carcinoembryonic antigen (CEA) have also been explored as targets. A trial of bronchoscopic delivery of CEA-specific CAR T-cells (NCT02349724) was initiated, but public updates on outcomes remain limited. A second trial (NCT06043466) targeting CEA in multiple solid tumors is currently recruiting.

Another promising target under investigation is MUC1, a mucin overexpressed and aberrantly glycosylated in NSCLC. A Phase I trial (NCT02587689) evaluated MUC1 CAR T-cell therapy in heavily pretreated NSCLC patients. Preliminary data suggested that MUC1 CAR T-cells could mediate cytotoxicity without overt off-tumor toxicity, though responses were predominantly disease stabilization.

Emerging strategies to improve CAR T efficacy in lung cancer include the development of “armored” CARs capable of secreting cytokines such as IL-12, incorporation of checkpoint blockade molecules directly into CAR T constructs, and targeting multiple antigens simultaneously to mitigate the risk of antigen escape. The use of bispecific CARs, for example, targeting EGFR and B7-H3, is being investigated in preclinical models and early-phase trials [50,51].

Toxicities observed in lung cancer CAR T trials have largely been manageable, with low rates of severe CRS or neurotoxicity. However, concerns regarding on-target/off-tumor toxicity, given the expression of many TAAs on normal epithelial tissues, necessitate careful selection and engineering of CAR T constructs. Table 3 summarizes the completed and ongoing CAR T-cell therapy clinical trials recruiting lung cancer patients.

### 9.2. Small Cell Lung Cancer (SCLC)

Small cell lung cancer (SCLC) is a highly aggressive form of lung cancer, characterized by rapid growth, early metastasis, and poor prognosis. Despite the initial effectiveness of chemotherapy and radiation therapy, SCLC almost invariably relapses and remains highly resistant to treatment in its advanced stages. The introduction of immunotherapies, including immune checkpoint inhibitors, has not significantly improved long-term survival in SCLC, which underscores the urgent need for novel therapeutic strategies. CAR T-cell therapy, particularly its potential for targeting tumor-specific antigens, represents a promising direction for treating this aggressive malignancy.

#### 9.2.1. Targeting Neuroendocrine Markers in SCLC

SCLC is primarily composed of neuroendocrine cells that often express specific markers such as CD56, ASCL1 (Achaete-Scute Family BHLH Transcription Factor 1), and the neural cell adhesion molecule (NCAM), also known as CD56. These markers provide potential targets for CAR T-cell therapies in SCLC. Neuroendocrine differentiation in SCLC is associated with aggressive tumor biology and targeting these markers through CAR T-cell therapy has gained significant interest [52,53,54,55].

One of the most studied targets in CAR T-cell therapy for SCLC is CD56, a well-established neuroendocrine marker. Preclinical studies demonstrated that CD56 CAR T-cells exhibited potent cytotoxicity against CD56-positive tumor cell lines in vitro and significantly suppressed tumor growth in xenograft models. Importantly, the study also addressed potential off-target toxicity by evaluating the effects of CD56 CAR T-cells on normal tissues, which express CD56 at lower levels. The findings support the feasibility of targeting CD56 as a therapeutic strategy in neuroendocrine tumors [55].

A major concern with CD56-targeted CAR T-cell therapy is the potential for off-tumor toxicity, as CD56 is expressed not only on SCLC cells but also on normal tissues, including neurons. This risk raises the possibility of neurotoxicity, which has been observed in other CAR T-cell trials targeting CD56. Thus, further optimization of CAR T-cell doses and improvements in targeting specificity are necessary to minimize collateral damage to normal tissues.

#### 9.2.2. Targeting DLL3 in SCLC

Delta-like ligand 3 (DLL3) is another promising target in SCLC due to its selective expression on SCLC cells and its minimal expression on normal adult tissues. DLL3 is a Notch pathway ligand that is implicated in the regulation of tumor growth and differentiation. Overexpression of DLL3 has been observed in most SCLC cases, making it an attractive target for CAR T-cell therapy [56].

Few clinical trials are currently investigating DLL3-targeted CAR T-cells in SCLC either alone or in combination with PD-L1 inhibitors (NCT05680922, NCT06348797).

The neurotoxicity associated with DLL3-targeted CAR T-cells is of particular concern, given the expression of DLL3 in the central nervous system and its role in neuronal differentiation. To mitigate these risks, some strategies include pre-treatment with immunosuppressive agents or modified CAR T-cells designed to reduce the likelihood of neurotoxic effects.

Overall, CAR T-cell therapy for lung cancer remains in early development, with most studies limited to Phase I trials. Antigen selection, toxicity mitigation, and trafficking remain key challenges in translating preclinical promise to clinical benefit.

## 10. CAR T-Cell Therapy in Colorectal Cancer

Colorectal cancer (CRC) remains one of the most prevalent malignancies worldwide, with high mortality, particularly in metastatic disease. Standard therapeutic strategies, including chemotherapy, targeted agents (such as anti-EGFR antibodies), and immune checkpoint inhibitors, have offered incremental gains. However, immunotherapies have been largely limited to microsatellite instability-high (MSI-H) tumors, leaving the majority of microsatellite stable (MSS) CRCs without effective immune-based options. In this context, CAR T-cell therapy is under investigation as a novel immunotherapeutic strategy, though its application has been hindered by challenges, including target selection, tumor immune evasion, and the immunosuppressive TME.

Several tumor-associated antigens have been explored as targets for CAR T-cell therapy in CRC. One of the earliest and most extensively studied is carcinoembryonic antigen (CEA), a glycoprotein overexpressed in the majority of CRC tumors but also found at lower levels in normal colonic and pulmonary tissues [57]. Several studies are currently recruiting patients.

In a Phase I, open-label, dose-escalation trial (NCT05396300), the safety and efficacy of a novel hypoxia-responsive CEA-targeted CAR T-cell therapy were evaluated in 40 patients with advanced solid tumors refractory to standard treatments, including colorectal (*n* = 35), gastric (*n* = 3), NSCLC (*n* = 1), and biliary tract cancer (*n* = 1). Patients received either intraperitoneal (I.P., *n* = 16) or intravenous (I.V., *n* = 24) infusions at low (1–3 × 10^6^ CAR^+^/kg) or high (4–6 × 10^6^ CAR^+^/kg) doses. Grade 1–2 CRS was observed in 62.5% of patients. Two patients developed severe colitis accompanied by profuse diarrhea following CAR T-cell therapy. Standard anti-inflammatory treatments proved ineffective, and both patients experienced cascading complications. These cases underscore the potential gastrointestinal toxicity of the therapy and prompt further investigation into underlying mechanisms and risk mitigation strategies [58]. The I.P. group showed superior efficacy, with an ORR of 25% versus 8% in the I.V. group and a DCR of 88% vs. 67%, respectively. In the high-dose I.P. subgroup, the ORR increased to 28.6%, with sustained tumor remission beyond 5 months in responders, highlighting I.P. administration as a promising route for CEA CAR T delivery in solid tumors [59].

A subsequent trial (NCT03682744) utilized intraperitoneal administration of CEA CAR T-cells for peritoneal carcinomatosis from CRC. This localized approach appeared safer, with limited systemic toxicity and some evidence of tumor cytoreduction on imaging, although durable responses were rare. Cytokine release syndrome (CRS) was infrequent and limited to grade 1–2. This trial is currently withdrawn due to enrollment challenges.

In a Phase I, dose-escalation clinical trial (NCT02349724), CEA-directed CAR T-cell therapy was evaluated in patients with metastatic CRC. Of the ten patients treated, seven experienced disease stabilization (up to 30 weeks), and two showed partial tumor responses by imaging criteria [60]. Notably, the therapy was well tolerated, with no treatment-related adverse events reported. A separate Phase Ib study (NCT02416466) investigated the safety and feasibility of intra-arterial delivery of anti-CEA CAR T-cells in combination with selective internal radiation therapy in six patients with colorectal liver metastases. The regimen was well tolerated, with no grade 4 or 5 toxicities and no cases of severe cytokine release syndrome or neurotoxicity observed. The median overall survival was reported at 8 months, suggesting a favorable safety profile and potential clinical benefit in this heavily pretreated population [61].

In a single-arm, dose-escalation Phase I trial (NCT05240950), anti-CEA CAR T-cells were administered to patients with colorectal cancer liver metastases (CRLM) who had no evidence of disease post-treatment and ≥ 30% CEA expression. Twelve patients received CAR T infusions across three dose levels (1, 3, and 6 × 10^6^/kg), with safety and relapse-free survival (RFS) as primary endpoints. No severe adverse events were reported; mild toxicities included lymphopenia, arthralgia, fever, and rash. Among nine relapse-free patients prior to infusion, five experienced recurrences during a median follow-up of 23 months. In the highest-dose group, 57.14% (4/7) remained relapse-free at 5, 7, 10, and 15 months post-infusion, suggesting a potential benefit in delaying recurrence. This is the first clinical trial to demonstrate the feasibility and safety of adjuvant anti-CEA CAR T-therapy in post-resection CRLM, with promising signals of prolonged RFS at higher dosing levels [62].

Guanylyl cyclase C (GUCY2C), a receptor expressed almost exclusively on intestinal epithelial cells and retained in metastatic CRC, has also emerged as a promising target [63]. Two Phase I trials (NCT04652219 and NCT04780529) are currently evaluating GUCY2C-directed CAR T-cells in patients with advanced CRC. Eligible participants require biopsy-confirmed GUCY2C expression. While no efficacy data have been reported to date, preclinical studies suggest minimal risk of off-tumor toxicity given GUCY2C’s restricted expression in the gut lumen, potentially allowing systemic administration without major adverse effects.

EpCAM, a cell adhesion molecule implicated in cancer stemness and metastatic potential, is another target under investigation [64]. A Phase I trial (NCT03013712) studied EpCAM-CAR T-cells in advanced gastrointestinal tumors, including CRC. Regional hepatic artery infusion was used to limit systemic exposure. Early-phase data confirmed safety and procedural feasibility, though anti-tumor efficacy was limited, likely due to low target density or immune evasion.

Emerging strategies to enhance CAR T efficacy in CRC include dual-targeted CAR T-cells designed to recognize multiple antigens simultaneously to reduce the risk of antigen escape. For example, bispecific CAR Ts targeting CEA and EpCAM or GUCY2C and HER2 are under preclinical development [65]. Furthermore, armored CAR T-cells engineered to secrete pro-inflammatory cytokines such as IL-12 or resist TGF-β-mediated suppression are entering early-phase trials [57,66,67].

The immunosuppressive microenvironment of CRC, particularly in MSS tumors, represents a major barrier. Tumor-infiltrating regulatory T-cells (Tregs), myeloid-derived suppressor cells (MDSCs), and high levels of TGF-β collectively impair CAR T persistence and functionality. Trials combining CAR T-therapy with immune checkpoint blockade (e.g., anti-PD-1 or anti-TGF-β antibodies) are underway to overcome these obstacles [57].

While no CAR T product has yet received approval for CRC, ongoing and future trials hold promise, particularly with the incorporation of multi-antigen targeting strategies, armored CAR designs, and improved trafficking to the TME. The field remains in early clinical phases, with Phase II trials (e.g., expanded CT041 studies for CLDN18.2-positive CRC) anticipated in the near future.

## 11. CAR T-Cell Therapy in Esophageal, Gastroesophageal, and Gastric Cancers

Gastric and gastroesophageal junction (GEJ) adenocarcinomas are among the most lethal solid tumors globally, often diagnosed at advanced stages with limited curative options. While immune checkpoint inhibitors have demonstrated some clinical benefit in PD-L1-positive tumors [68,69], overall survival remains poor, highlighting the unmet need for novel immunotherapeutic strategies such as CAR T-cell therapy, especially for CLDN18.2-positive subgroups.

One of the principal challenges in applying CAR T-cell therapy to gastric cancer is the identification of suitable tumor-specific antigens that are sufficiently overexpressed in malignant cells while sparing normal tissues. Several targets have emerged as promising candidates, leading to early-phase clinical trials.

Claudin 18.2 (CLDN18.2), a tight junction protein normally confined to gastric mucosa but aberrantly exposed and overexpressed in gastric and GEJ cancers [70], has been at the forefront of CAR T development.

A recent Phase II open-label, multicenter, randomized controlled trial was presented at ASCO 2025 (NCT04581473). This study was conducted in China and enrolled 156 patients with advanced gastric or gastroesophageal junction cancer positive for CLDN18.2 expression (≥ 2+ intensity, ≥ 40% tumor cells), refractory to at least two prior treatments [71]. Patients were randomly assigned 2:1 to receive either satri-cel, a cellular therapy infused up to three times at 250 million cells per dose, or treatment of physician’s choice (TPC), including standard agents like nivolumab and paclitaxel. Of those assigned, 85% in the satri-cel group and 92% in the TPC group received treatment. Satri-cel patients had a higher burden of prior therapy and peritoneal metastasis. After a median follow-up of 9.1 months (satri-cel) versus 3.5 months (TPC), median progression-free survival (PFS) was significantly longer with satri-cel (3.25 vs. 1.77 months; hazard ratio 0.37, *p* < 0.0001). Grade 3 or higher treatment-emergent adverse events were more frequent with satri-cel (99% vs. 63%), primarily hematologic toxicities such as decreased lymphocytes, white blood cells, and neutrophils. Cytokine release syndrome occurred in 95% of satri-cel recipients. Multi-omics analysis from this trial identified GZMK^+^ Tpex cells as key mediators of anti-tumor immunity and response. Resistance was linked to IQGAP3^+^ tumor cells and TGF-β pathway activation, highlighting potential therapeutic targets to optimize satri-cel efficacy.

Also, a single-arm, open-label, phase 1 trial (NCT03874897) evaluated the safety and efficacy of satricabtagene autoleucel (satri-cel), a CLDN18.2-targeted autologous CAR T-cell therapy, in 98 patients with advanced CLDN18.2-positive gastrointestinal cancers. The study included dose-escalation and expansion cohorts, with arms testing satri-cel alone or in combination with anti–PD-1 blockade. Patients received up to 5.0 × 10^8^ CAR T-cells, with a median follow-up of 32.4 months. The overall response rate was 38.8%, disease control rate 91.8%, median progression-free survival 4.4 months, and median overall survival 8.8 months. No dose-limiting toxicities, treatment-related deaths, or neurotoxicity were observed. Cytokine release syndrome occurred in 96.9% of patients (all grade 1–2), and gastric mucosal injuries in 8.2% [72].

Other antigens under investigation include HER2, MUC1, and CEA. HER2, overexpressed in a subset of gastric and GEJ adenocarcinomas, represents a well-validated target in antibody-based therapies such as trastuzumab. In this context, an ongoing HER2-targeted CAR T-cell therapy is being studied in a Phase I trial (NCT02713984). Eligible patients were required to have HER2-positive tumors (IHC 3+ or FISH amplified). Preliminary clinical outcomes have not yet been reported.

Mucin 1 (MUC1) is a transmembrane glycoprotein that is overexpressed and aberrantly glycosylated in various cancers, including gastric cancer. In tumor cells, MUC1 exhibits altered glycosylation patterns, leading to the exposure of novel epitopes that are not present in normal tissues, making it an attractive target for immunotherapy. A first-in-human Phase I trial (NCT03525782) evaluated MUC1-CAR T-cells in solid tumors, including gastric adenocarcinoma, demonstrating acceptable safety profiles and signs of disease stabilization in a subset of patients.

CEA-directed CAR T-therapies are similarly under evaluation. Several clinical trials are currently ongoing. Given CEA expression in normal gastrointestinal tissues, there is concern regarding on-target, off-tumor toxicity; due to CEA’s physiological expression in normal gastrointestinal tissues, off-tumor toxicity remains a key safety concern.

Overall, while no CAR T-therapy has yet achieved regulatory approval in gastric or gastroesophageal cancers, the field is advancing rapidly. CLDN18.2-directed CAR T-therapy, in particular, holds substantial promise and may potentially achieve breakthrough designation if current Phase II/III trials replicate the early efficacy signals.

## 12. CAR T-Cell Therapy in Urological Cancers

Urological malignancies, including bladder (urothelial), prostate, and renal cell cancers, present unique immunologic environments and expression profiles that influence the potential efficacy of CAR T-cell therapy [73]. While the field is relatively nascent, several preclinical and early-phase trials are exploring CAR T-cells against both lineage-restricted (e.g., PSMA) and broadly expressed tumor-associated antigens in urological cancers. Of particular interest are prostate-specific membrane antigen (PSMA), vascular endothelial growth factor receptor (VEGFR), and HER2, among others.

### 12.1. Renal Cell Carcinoma

Renal cell carcinoma (RCC), accounting for approximately 90% of all kidney malignancies, has shown significant responsiveness to immunotherapeutic approaches, including checkpoint inhibitors [74,75,76,77]. Given the immunogenic potential of RCC, adoptive cell therapies such as CAR T-cells have garnered interest.

CAIX (Carbonic Anhydrase IX) is a transmembrane enzyme induced by hypoxia that regulates pH in tumor cells, promoting survival, proliferation, and metastasis [78].

#### 12.1.1. Target Antigens and CAR Constructs

Preclinical studies have shown that CAR T-cells targeting CAIX, c-MET, and CD70 exhibit significant anti-tumor activity in xenograft models. Synergistic effects have also been demonstrated when combining CAR T-cells with agents such as sunitinib, axitinib, or oncolytic adenoviruses, suggesting the potential of multimodal strategies [79,80,81,82]. However, clinical translation has been challenging. For instance, trials targeting VEGFR2 and CAIX have shown limited efficacy or off-tumor toxicities, underscoring the critical importance of careful antigen selection in solid tumors [83]. Several tumor-associated antigens (TAAs) have been explored in CAR T trials targeting RCC. Among these, carbonic anhydrase IX (CAIX) has been the most extensively investigated. CAIX is highly expressed in over 90% of clear cell RCC (ccRCC) tumors and shows limited expression in normal tissues, making it a compelling immunotherapy target [84]. A few ongoing clinical trials are investigating CAIX-targeting CAR T-cell therapies in RCC.

Two foundational clinical trials targeting CAIX with CAR T-cell therapy in metastatic clear cell RCC (ccRCC) were conducted using G250/CAIX-specific single-chain variable fragment (scFv) fused to CD3ζ (first generation). In the initial Phase I study, twelve patients received escalating doses (0.2–2.1 × 10^9^ CAR T-cells). Although CAR T-cells trafficked to tumors, half of the patients experienced grade 2–4 hepatobiliary toxicity due to CAIX expression on bile duct epithelium, identified on liver biopsy, leading to treatment cessation [85].

A follow-up protocol pre-treated four patients with the CAIX-targeting monoclonal antibody G250 to block CAIX in normal tissues. This pre-treatment prevented hepatotoxicity and improved CAR T-cell persistence, yet tumor regression remained minimal, with no objective responses despite evidence of biological activity [86].

#### 12.1.2. CD70 as an Emerging Target

CD70 is a TNF ligand overexpressed in most ccRCC tumors, with limited normal tissue expression. Its interaction with CD27 drives immune evasion and T-cell exhaustion, making it a rational CAR T target [87]. In ccRCC, CD70 expression correlates with immune evasion, T-cell exhaustion, and poor prognosis. Its interaction with the CD27 receptor promotes T-cell apoptosis and regulatory T-cell expansion within the TME [88]. Given its selective expression and functional role in immune suppression, CD70 is a compelling target for CAR T-cell therapy. CAR T-cells engineered to target CD70 can directly eliminate tumor cells while potentially reversing immune suppression. Preclinical studies have demonstrated robust CD70-directed cytotoxicity, providing a strong rationale for clinical translation in RCC [89].

CTX130 is an investigational allogeneic CD70-targeted CAR T-cell therapy developed using CRISPR-Cas9 gene editing. It was evaluated in the phase 1 COBALT-RCC trial (NCT04438083) for patients with advanced clear cell renal cell carcinoma (ccRCC) who had previously received checkpoint inhibitors and/or VEGF-targeted therapies. The trial demonstrated a favorable safety profile, with no dose-limiting toxicities, immune effector cell-associated neurotoxicity syndrome, or graft-versus-host disease. CRS occurred in 50% of patients, all grade 1 or 2. Notably, one patient achieved a durable complete response lasting over three years, marking the first such response in an allogeneic CAR T-cell therapy for solid tumors. Overall, 81.3% of patients experienced disease control, including 75% with stable disease. These findings underscore the potential of CTX130 as a promising treatment for ccRCC and other CD70-positive malignancies [90].

ALLO-316 is an allogeneic CAR T-cell therapy derived from healthy donors, engineered to target CD70-expressing tumor cells and CD70-positive host T-cells that may mediate rejection. In the phase 1a/b TRAVERSE trial (NCT04696731), patients with advanced clear cell renal cell carcinoma (ccRCC) who had progressed after immune checkpoint inhibitors and VEGF-targeted treatments received a single infusion of ALLO-316 following lymphodepletion. Of 39 treated patients (82% CD70-positive), the therapy showed manageable safety with no graft-versus-host disease. Adverse events were common but generally manageable, with serious cytokine release syndrome (CRS) in only 2% of patients and no cases of grade ≥3 neurotoxicity. The overall response rate (ORR) was 20%, with a confirmed ORR of 33% in patients whose tumors expressed high levels of CD70 (≥50%), and all responses were ongoing at the time of data cut-off. These results support continued investigation of ALLO-316 in CD70-positive ccRCC [91]. Table 4 summarizes the completed and ongoing CAR T-cell therapy clinical trials recruiting renal cell carcinoma patients.

## 13. Prostate Cancer

Prostate cancer has been a prominent target in the urologic space for CAR T development due to the relatively restricted expression of PSMA on malignant epithelial cells.

Prostate cancer (PCa) expresses several tumor-specific surface antigens that are well-suited for targeted immunotherapies, particularly CAR T-cell approaches. Two of the most extensively studied antigens are prostate-specific membrane antigen (PSMA) and prostate stem cell antigen (PSCA). PSMA is a type II transmembrane glycoprotein predominantly expressed on the surface of prostatic epithelial cells and is markedly upregulated in prostate cancer tissues and tumor neovasculature, while remaining minimally expressed in normal tissues such as the intestine, liver, and kidney [92]. PSCA is another promising tumor-associated antigen, detected primarily on the membrane of prostate cancer cells with significantly higher expression in malignant compared to normal prostate tissue, and absent in other healthy tissues [93]. This high tumor-specific expression profile and limited off-tumor activity make both PSMA and PSCA attractive targets, with a favorable safety profile based on preclinical and early-phase studies.

One of the leading candidates is PSMA-directed CAR T-therapy. CAR T-PSMA-02 (NCT04227275) was a Phase 1 trial evaluating PSMA-targeted CAR T-cells co-engineered with a dominant-negative TGFβ receptor (PSMA-CART-TGFβRDN) to counteract immunosuppression in patients with metastatic castration-resistant prostate cancer (mCRPC). CRS was mild (grade 1–2) in all evaluable patients, though two patients experienced dose-limiting toxicities, including one case of fatal neurotoxicity and multi-organ failure, consistent with macrophage activation syndrome. Preliminary efficacy showed stable disease in 4 of 5 evaluable patients, with PSA declines ≥50% in 2 of 5 patients. One patient experienced fatal neurotoxicity consistent with macrophage activation syndrome [94].

A Phase I clinical trial (NCT03089203) evaluated PSMA-targeted CAR T-cells in patients with castration-resistant prostate cancer. Among the 13 participants, five experienced grade 2 cytokine release syndrome, while three showed a ≥30% reduction in PSA levels. These findings demonstrate that the clinical application of PSMA-directed CAR T-cells is feasible and generally well tolerated [95]. Nonetheless, the anti-tumor efficacy of CAR T-therapy in prostate cancer requires further improvement.

## 14. Bladder Cancer

Despite advances in the treatment of muscle-invasive bladder cancer (MIBC), and urothelial cancers in general, including the development of antibody-drug conjugates like enfortumab vedotin and the integration of immunotherapy with chemotherapy, the mortality rate remains high, especially in metastatic cases [96,97]. Consequently, there is a pressing need for novel therapeutic approaches such as CAR T-cell therapy. Urothelial carcinoma presents greater antigen heterogeneity, such as PSMA, HER2, EGFR, and MUC1 and an immune-evasive microenvironment. As such, CAR T-cell therapy holds promise for bladder cancer. There are a few ongoing clinical trials evaluating CAR T-cell therapies targeting HER2, PSMA, and ROR2, but published data on safety and efficacy are currently limited.

## 15. CAR T-Cell Therapy in Gynecological Cancers

Gynecological cancers, including ovarian, uterine, and cervical cancers, represent a significant clinical challenge due to their high mortality rates and often late-stage diagnosis. While conventional treatments such as surgery, chemotherapy, and radiation therapy have advanced over the years, the recurrence of metastatic disease remains a major hurdle, particularly in high-risk subgroups. Immunotherapy, including CAR T-cell therapy, is emerging as a promising new approach to target these malignancies, though several obstacles remain, including the identification of specific tumor-associated antigens and overcoming the immunosuppressive microenvironment.

### 15.1. Ovarian Cancer

Ovarian cancer is the leading cause of gynecological cancer-related deaths, with a poor prognosis due to late-stage presentation and frequent recurrence after first-line chemotherapy. High-grade serous ovarian carcinoma (HGSC), which accounts for the majority of ovarian cancer cases, has been the most common focus of CAR T-cell therapy trials.

B7-H3 (CD276) is an immunoregulatory molecule of the B7 family, minimally expressed in normal tissues but highly overexpressed in many solid tumors, including ovarian cancer. This tumor-specific pattern makes it an appealing immunotherapy target. B7-H3 promotes tumor progression by inhibiting T-cell activation, facilitating immune evasion, and enhancing proliferation, invasion, and metastasis. Its dual role as both an immune checkpoint and oncogenic driver underscores its potential as a priority target for CAR T-cell development [98].

Preclinical data indicate that B7 H3-directed CAR T-cells demonstrated potent, antigen-dependent anti-tumor activity in ovarian cancer models. These CAR T-cells effectively eliminated B7 H3-positive ovarian cancer cells both in vitro and in orthotopic/metastatic xenograft mouse models, including patient-derived tumors, significantly reducing tumor burden and improving survival without apparent toxicity [99]. Several clinical trials (e.g., NCT05211557, NCT06646627, NCT03585764) are now evaluating the safety and efficacy of B7-H3 CAR T-therapies in solid tumors, including ovarian cancer. These trials incorporate design innovations such as dual-targeting CAR constructs, suicide switches, and armored CAR T-cells to overcome immunosuppressive mechanisms in the ovarian TME. As this approach matures, B7-H3 CAR T-cells may emerge as a cornerstone therapy for heavily pretreated ovarian cancer patients, offering a potential option for patients refractory to conventional therapies.

CD70 is a transmembrane ligand overexpressed in several solid tumors (including ovarian cancer), with minimal presence on normal tissues. CD70 interacts with CD27 on T-cells to promote tumor proliferation, survival, and immune suppression via the NF-κB and PI3K pathways [100]. Exploiting CD70’s tumor-specific expression and immunosuppressive role, early studies have evaluated CD70-targeted CAR T-therapies in gynecologic cancers including ovarian cancer. Notably, a few Phase I trials (e.g., NCT05518253, NCT06215950, NCT05420545, NCT05468190) are investigating the safety and efficacy of CD70 CAR T-cells in advanced/metastatic solid tumors, including epithelial ovarian cancer. While detailed ovarian-specific preclinical results from this trial are still emerging, broader early-phase data show that CD70 CAR T-cells can induce meaningful anti-tumor activity in solid malignancies, and the therapy is being refined with strategies like allogeneic “off-the-shelf” constructs and safety switches (e.g., TCR or CD52 knockouts and safety mimotopes) to mitigate toxicity and improve access.

Targeting folate receptor-alpha (FRα), a cell surface protein overexpressed in many ovarian cancers, has shown promise. A Phase I trial (NCT03585764) investigated the safety and feasibility of FRα-targeted CAR T-cells in patients with relapsed or refractory ovarian cancer. However, the trial has been stopped due to recruitment barriers.

In addition to the above targets, mesothelin is another antigen of interest in ovarian cancer. Mesothelin is overexpressed in ovarian cancer and associated with poor prognosis. A few clinical studies (NCT05568680, NCT03799913, NCT04503980) are underway to investigate mesothelin-targeted CAR T-cells in patients with advanced ovarian cancer. Early data have demonstrated that mesothelin-directed CAR T-cells can mediate tumor lysis, although CRS was noted in a small number of patients.

Additionally, ongoing clinical trials targeting Claudin18.2, MUC16, and MUC1 are also recruiting patients with advanced ovarian cancer.

### 15.2. Uterine Cancer

Uterine cancer, including both endometrial and uterine serous carcinoma, presents unique challenges for CAR T-cell therapy. Uterine cancer is often associated with mutations in key tumor suppressor genes such as PTEN and TP53, leading to alterations in tumor biology that complicate treatment. Few CAR T studies have focused on uterine cancer. Ongoing efforts aim to identify tumor-specific antigens such as MUC16 and incorporate immunomodulatory strategies to overcome resistance.

### 15.3. Cervical Cancer

Cervical cancer, driven primarily by human papillomavirus (HPV) infections, remains a significant public health issue, especially in regions with low screening rates [101]. Despite the advent of HPV vaccines, the treatment of advanced cervical cancer still relies heavily on chemotherapy and radiation, with limited immunotherapeutic options available [102]. CAR T-cell therapy, particularly targeting HPV-related antigens, offers potential, though much work is needed to optimize these approaches [103].

CARs engineered to recognize HPV oncoproteins, particularly E6 and E7, have demonstrated potent preclinical efficacy [104]. Notably, a novel class of CARs based on T-cell receptor mimic (TCRm) nanobodies directed against HPV16 E6 exhibited robust anti-tumor responses in cervical cancer models, including activity against tumors with low antigen density and resistance to T-cell exhaustion [104]. Similarly, studies using optimized CAR constructs with selective affinity and functional persistence against HPV-positive cells reported potent and specific cytotoxicity in vitro and in vivo [105]. These findings complement earlier work demonstrating clinical responses with TCR-engineered T-cells targeting HPV E6 in refractory cervical cancer [106]. Collectively, these data support the continued development of CAR T-cell strategies, including TCR mimic and “armored” constructs, as a promising therapeutic frontier for HPV-driven malignancies.

A first-in-human Phase I trial (NCT03578406) is evaluating TCR-engineered T-cells against HPV E6 in patients with advanced cervical or head and neck cancers. Although focused on TCR rather than CAR T-cells, this trial demonstrates early feasibility and safety of antigen-specific T-cell therapy in HPV-positive malignancies. Preclinical data further show that engineered T-cells against E7 can drive tumor regression in vivo [107]. Together, these findings support advancing CAR T-cell approaches targeting E6 and E7 in cervical cancer, emphasizing the need for continued clinical development and optimization.

In addition to HPV-targeted CAR-Ts, CD70, mesothelin, and CEA have also been explored in cervical cancer. Several ongoing Phase I trials (NCT06215950, NCT06010875, NCT02961829) are testing CAR T-cells with the above targets in cervical cancer. Early-stage results indicate that the therapy is well tolerated, with CRS being manageable, but response rates have been limited. Combination regimens, including checkpoint inhibitors or chemotherapy, are being evaluated to enhance the modest efficacy of mesothelin-directed CAR T-cells in cervical cancer.

The major challenge in CAR T-cell therapy for cervical cancer, as with other gynecological cancers, is the immunosuppressive TME. High levels of TGF-β, regulatory T-cells (Tregs), and myeloid-derived suppressor cells (MDSCs) create a hostile environment that limits CAR T-cell expansion and function [108,109,110]. To address this, researchers are developing armored CAR T-cells engineered to secrete cytokines such as IL-12 or IL-15 to counteract the immunosuppressive signals within the TME [111,112].

## 16. CAR T-Cell Therapy in Head and Neck Cancers

Head and neck cancers (HNC) encompass a heterogeneous group of tumors that arise in the oral cavity, pharynx, larynx, and other structures of the head and neck. These cancers are often diagnosed at advanced stages, and despite aggressive treatment with surgery, radiation, and chemotherapy, the prognosis remains poor for patients with recurrent or metastatic disease [113]. The advent of immunotherapy, including CAR T-cell therapy, has opened new possibilities, particularly for those with HPV-negative cancers, which remain difficult to treat with conventional immune checkpoint inhibitors [114].

### 16.1. Targeted Approaches in Head and Neck Cancer

Several tumor-associated antigens are being explored for CAR-T cell therapy in head and neck cancers. HER2-directed CAR T-cells induced a 56% reduction in tumor size, aligning with their association with poor prognosis in HNSCC [115]. EGFR-targeted CAR T-cells enhanced cytokine secretion and cytolysis in EGFR-positive hypopharyngeal carcinoma models [116]. CD70-targeted CAR T-cells effectively eliminated HNSCC cells, reflecting CD70’s link to immunosuppression [117]. MUC1-directed CAR T-cells engineered to secrete IL-22 significantly suppressed tumor growth in vivo [118]. CD44v6-specific CAR T-cells achieved near-complete cytotoxicity against CD44v6+ tumor lines [119].

Multiple ongoing clinical trials are investigating CAR T-cell therapies targeting a variety of antigens in head and neck squamous cell carcinoma, a challenging solid tumor type. One approach is T4 immunotherapy, which engineers autologous T-cells to express dual receptors targeting ErbB dimers, commonly overexpressed in HNSCC, and promoting T-cell proliferation through key signaling pathways. Early-phase trials of T4 therapy have demonstrated promising anti-tumor activity with favorable safety profiles, including a notable disease control rate of 69% and minimal toxicity, especially when combined with immune checkpoint inhibitors such as nivolumab [NCT01818323] [120]. Other CAR T targets under clinical evaluation include EGFR [NCT02980315], MUC1 [NCT03013712], CD44v6 [NCT04729543, NCT04097301], PD-L1 [NCT05117138], and CSPG4 [NCT06096038], reflecting efforts to overcome tumor heterogeneity and immune evasion. These studies also highlight ongoing challenges such as limited CAR T-cell persistence, TME immunosuppression, and on-target/off-tumor toxicity.

Several trials are exploring innovative strategies to improve CAR T-cell efficacy and safety in HNSCC, such as intratumoral CAR T-cell delivery to reduce systemic toxicity, hypoxia-sensing CARs to enhance tumor selectivity, and combination therapies incorporating immune checkpoint blockade or chemotherapy. Additionally, newer platforms, including CAR-engineered γδT cells [NCT04107142] and natural killer (CAR-NK) cells [NCT04847466], as well as RNA vaccine combinations targeting CLDN6 [NCT05147649], are being assessed to boost anti-tumor responses. The role of lymphodepletion before CAR T-therapy remains uncertain for solid tumors like HNSCC, with evidence suggesting it may not be essential in all cases. Overall, while CAR T-cell therapy in HNSCC is still at an early clinical stage, the encouraging preliminary data support further development to optimize dosing, target selection, and combination strategies for improved patient outcomes.

### 16.2. HPV-Positive Head and Neck Cancer

Similarly to cervical cancer studies, human papillomavirus-related head and neck cancers, particularly oropharyngeal cancers, have a more favorable prognosis than HPV-negative cancers, largely due to better response rates to immunotherapy. HPV-related cancers express HPV-specific antigens, such as the E6 and E7 oncoproteins, which are ideal targets for CAR T-cell therapy.

CAR T-cell therapy for head and neck squamous cell carcinoma (HNSCC) faces significant hurdles primarily due to tumor antigen heterogeneity and the immunosuppressive TME. Tumors in HNSCC exhibit diverse antigen profiles, including neoantigens, viral antigens (notably in HPV-related cases), and heterogeneous tumor-associated antigens, which can lead to immune escape as some tumor cells lose or downregulate target antigens, limiting CAR T-cell effectiveness. Additionally, off-target toxicities are a concern.

### 16.3. Future Directions in Head and Neck Cancer CAR T-Cell Therapy

Looking ahead, the integration of CAR T-cell therapy with other immunotherapeutic approaches is a major area of focus. For example, PD-1- or CTLA-4-based inhibitory chimeric antigen receptors may offer a way forward to overcome the off-target toxicities of CAR T-cell therapies [121]. The combination of CAR T-cells with radiation therapy is also being investigated to enhance CAR T-cell efficacy by inducing immunogenic cell death and promoting antigen release [122].

## 17. CAR T-Cell Therapy in Pancreatic and Hepatobiliary Cancers

Pancreatic ductal adenocarcinoma (PDAC) and hepatobiliary malignancies, including hepatocellular carcinoma (HCC) and cholangiocarcinoma, represent some of the most challenging solid tumors for immunotherapy, largely due to their dense stromal barriers, immunosuppressive TME, and low tumor mutational burden. Despite these challenges, preclinical studies have shown that CAR T-cell therapy can mediate anti-tumor responses in these settings when appropriately engineered to overcome immune exclusion and exhaustion. Several tumor-associated antigens have been evaluated as targets in PDAC, with mesothelin emerging as one of the most extensively studied. In murine models, mesothelin-targeted CAR T-cells demonstrated robust tumor eradication, especially when combined with lymphodepleting chemotherapy or engineered to co-express immune stimulatory molecules such as interleukin-12 (IL-12) or dominant-negative TGF-β receptors to enhance CAR T-cell function within the hostile pancreatic TME [123,124,125].

Beyond mesothelin, additional targets such as prostate stem cell antigen (PSCA), carcinoembryonic antigen (CEA), mucin-1 (MUC1), and CD24 have shown efficacy in preclinical models of pancreatic cancer. Notably, CD24-targeted CAR T-cells induced significant tumor regression in orthotopic PDAC mouse models and showed synergy with anti-PD-1 therapy [126]. CEA-targeted CAR T-cells, which had previously raised concerns over on-target/off-tumor toxicity in colorectal models, demonstrated anti-tumor efficacy when regional delivery strategies were employed to limit systemic exposure [127]. PSCA-directed CAR T-cells have also shown promising cytotoxicity against PSCA-expressing pancreatic tumors, and their efficacy was improved when combined with agents that remodel the stroma or disrupt immune checkpoints [128]. Trafficking of CAR T-cells into fibrotic tumor beds remains a significant hurdle in PDAC, and the incorporation of chemokine receptors (e.g., CCR2b, CXCR2) into CAR constructs has improved homing to tumor sites in murine models [129].

Clinical translation of these preclinical advances is underway, albeit cautiously. A first-in-human Phase I study (NCT01897415) of mesothelin-targeted CAR T-cells in patients with advanced PDAC demonstrated a favorable safety profile and some disease stabilization, although persistence of CAR T-cells was limited [130]. A separate trial (NCT03323944) exploring intraperitoneal administration of mesothelin CAR T-cells aims to overcome delivery challenges and enhance tumor penetration [131]. CEA-targeted CAR T-cells are also under investigation in PDAC and biliary cancers in early-phase trials (e.g., NCT02744287), utilizing both systemic and hepatic artery infusion to localize therapy while minimizing off-tumor effects [132]. Trials investigating PSCA- and MUC1-targeted CAR T-cell therapies (NCT02744287, NCT05239143) are ongoing, though published data remain limited.

In hepatobiliary cancers, especially HCC, glypican-3 (GPC3) is a promising antigen due to its high tumor specificity and minimal normal tissue expression. GPC3-targeted CAR T-cells have demonstrated anti-tumor activity in xenograft models and have now entered clinical testing. In a Phase I study (NCT03084380), GPC3-CAR T-cells were well tolerated, and some patients experienced disease stabilization or minor responses [133]. Other studies are investigating combination approaches, such as GPC3-CAR T-cells with sorafenib or checkpoint inhibitors to augment efficacy (NCT05003895). Meanwhile, CEA- and EpCAM-directed CAR T-cell therapies are being explored in cholangiocarcinoma, with ongoing trials (NCT02850536, NCT03633773) evaluating regional administration to overcome the anatomical complexity of the biliary tree. Despite these advances, challenges such as short CAR T-cell persistence, immune evasion mechanisms, and liver-specific toxicities necessitate continued optimization of both targets and delivery strategies.

## 18. CAR T-Cell Therapy in Sarcomas

We previously discussed the recent approval of cell therapy in synovial sarcoma. Sarcomas represent a heterogeneous group of mesenchymal tumors that can arise in any tissue type, including bone, muscle, fat, and connective tissues. These tumors can be classified into soft tissue sarcomas (STSs) and bone sarcomas, each with distinct clinical behaviors, molecular profiles, and therapeutic challenges. Despite improvements in surgical techniques and chemotherapy regimens, treatment outcomes for sarcomas remain unsatisfactory, particularly in metastatic or recurrent cases. CAR T-cell therapy holds promise for these malignancies, though significant challenges persist, including antigen specificity, tumor heterogeneity, and the need for effective target identification.

Beyond synovial sarcoma, other soft tissue sarcomas have also been explored in CAR T-cell therapy trials, though progress has been more limited. For example, targeting GD2, a disialoganglioside commonly expressed on several types of sarcomas, has been an area of interest. GD2 is notably overexpressed in neuroblastoma and osteosarcoma, both of which are sarcomas that present significant challenges in treatment [134,135]. A Phase I trial (NCT02107963) tested GD2-targeted CAR T-cells in patients with solid cancers, including osteosarcoma and rhabdomyosarcoma. The trial was designed to assess the safety and efficacy of GD2-CAR T-cells in these cancers, with an emphasis on reducing off-tumor toxicity while maintaining tumor-specific targeting.

Results from this trial demonstrated that GD2-CAR T-cells could effectively target tumor cells in patients with GD2-positive sarcomas. The primary adverse effect was CRS, which was manageable in most patients. While the overall response rate was modest, with some patients experiencing stable disease, the data suggested that GD2-targeted CAR T-cells could have therapeutic potential, particularly when combined with other modalities like cytokine therapy or immune checkpoint inhibitors to enhance CAR T-cell persistence and activity [136].

In a Phase 1 trial (NCT00902044), Hegde et al. evaluated the safety and early efficacy of autologous HER2-specific CAR T-cell therapy in advanced sarcoma patients following lymphodepletion. Thirteen patients were treated. The regimen was well tolerated: most experienced only mild to moderate (Grade 1–2) cytokine release syndrome, though two cases of severe CRS occurred at higher dose levels. Clinical benefit was observed in 50% of patients [137]. Spatial profiling of tumor biopsies revealed immune cell distribution differed by sarcoma subtype and therapeutic response. These findings confirm HER2 as a promising CAR T target in sarcoma and support further investigation of this approach.

Several other targets of interest such as insulin-like growth factor receptor 1 (IGF-1R), receptor tyrosine kinase-like orphan receptor 1 (ROR1), and Ephrin type-A receptor 2 (EphA2) are overexpressed in various sarcomas and few CAR T-cell therapy clinical studies are currently ongoing.

## 19. CAR T-Cell Therapy for Melanoma

Despite the dramatic successes of immune checkpoint inhibitors targeting CTLA-4 and PD-1/PD-L1 pathways, a significant proportion of patients experience disease progression or relapse [138]. Consequently, alternative immunotherapeutic strategies, including adoptive cell therapies such as TILs and CAR T-cells, are being actively investigated in melanoma. In February 2024, Lifileucel (Amtagvi), an autologous tumor-infiltrating lymphocyte (TIL) therapy, received FDA accelerated approval for adults with unresectable or metastatic melanoma who progressed following PD-1 inhibitor therapy—and, if harboring a BRAF V600 mutation, after BRAF ± MEK inhibitors [139]. Approval was based on results from the global, multicenter Phase II C-144-01 trial (NCT02360579). Patients underwent non-myeloablative lymphodepleting chemotherapy (cyclophosphamide plus fludarabine), received a single infusion of lifileucel (median dose 21.1 × 10^9^ viable TILs; range 7.5–72 × 10^9^), and up to six doses of high-dose interleukin-2. In the primary efficacy population of 73 evaluable patients, lifileucel achieved an objective response rate of 31.4% (95% CI: 24.1–39.4%), including complete and partial responses, with a rapid median time to response of approximately 1.4 months. Durability was notable: at five-year follow-up of 153 patients, the median duration of response reached 36.5 months, with approximately 31% of responders maintaining ongoing responses at five years, and median overall survival was 13.9 months (5-year survival rate ~19.7%) [140].

Multiple early-phase clinical trials have been launched, employing innovative CAR designs to enhance specificity, persistence, and anti-tumor activity.

Several melanoma-associated antigens have been investigated as targets for CAR T-therapy, each with distinct expression profiles and immunobiological implications. A promising antigen, interleukin-13 receptor alpha 2 (IL13Rα2), is overexpressed in melanoma and linked to tumor invasiveness and poor prognosis; IL13Rα2-targeted CAR T-cells are currently being studied in a Phase I clinical trial (NCT04119024).

Another area of interest is surface-expressed tyrosinase-related protein 1 (TYRP1), a melanosome-associated protein trafficked to the plasma membrane in cutaneous and rare melanoma subtypes. In vitro and in vivo studies using murine and patient-derived models of cutaneous, acral, and uveal melanoma demonstrated robust, antigen-specific cytotoxicity, cytokine release, and tumor regression, without off-tumor toxicity or systemic adverse effects in immunocompetent mouse models [141]. TYRP1 was highly overexpressed (log2 FPKM ≥ 7) in ~30% of melanomas overall and up to ~90% in uveal melanoma, including in biopsies from patients resistant to immune checkpoint inhibitors, supporting its clinical relevance. Based on safety and efficacy observed in preclinical models, the authors are preparing to initiate a first-in-human Phase I trial targeting TYRP1 in melanoma.

## 20. CAR T-Cell Therapy for Glioblastoma

Glioblastoma (GBM) remains the most aggressive and lethal primary brain tumor, characterized by rapid proliferation, diffuse infiltration, robust neovascularization, and profound resistance to conventional therapies. Despite maximal surgical resection, radiotherapy, and temozolomide chemotherapy, the median survival for GBM patients remains approximately 15–18 months [142]. Immunotherapy strategies, particularly checkpoint inhibitors, have yielded limited success, largely due to the profoundly immunosuppressive TME and the tumor’s extensive heterogeneity [143]. CAR T-cell therapy has emerged as a promising avenue for GBM, aiming to overcome the immune evasion mechanisms of the disease.

In a first-in-human, Phase I clinical trial (NCT05168423), researchers evaluated the safety and early efficacy of intracerebroventricular (ICV) delivery of bivalent CAR T-cells targeting EGFR epitope 806 and interleukin-13 receptor α2 (IL13Rα2) in 18 patients with recurrent, EGFR-amplified glioblastoma. The dose-escalation study determined a maximum tolerated dose of 2.5 × 10^7^ CAR T-cells, with no grade 4–5 neurotoxicity observed, although 56% of patients experienced grade 3 neurotoxic events. Of 13 evaluable patients, 8 (62%) showed tumor shrinkage. One had a confirmed partial response, and one maintained stable disease for over 16 months. Median progression-free survival was 1.9 months, and overall survival had not yet been reached at a median follow-up of 8.1 months [144].

The study confirms that locoregional administration of dual-targeted CAR T-cells, delivered directly into cerebrospinal fluid, can be performed safely in recurrent glioblastoma, achieving meaningful tumor engagement and early radiographic activity. While neurotoxicity was common, it remained manageable without any fatal events. The promising tumor responses, including durable disease control in select patients, highlight the therapeutic potential of multi-antigen CAR strategies and call for further dose expansion and mechanistic exploration in larger trials.

Several tumor-associated antigens have been studied, including the following:

B7-H3: The checkpoint molecule B7 H3 (CD276) is strongly expressed in gliomas, including DIPG. The Phase I BrainChild 03 trial (NCT04185038) investigated locoregional intracerebroventricular delivery of B7 H3-specific CAR T-cells. Among the first three evaluable pediatric DIPG patients, up to 40 infusions were administered without dose-limiting toxicity. Evidence of CAR T-cell persistence in CSF and signs of local immune activation were observed. The median post-infusion survival was 10.7 months, with a median overall survival of 19.8 months, some patients remained alive more than 50 months after diagnosis, spurring follow-up studies and FDA RMAT designation.

EGFRvIII, a GBM-specific oncogenic variant, has been targeted in two clinical approaches. The intravenous EGFRvIII-CAR trial (NCT02209376) showed tumor localization and antigen loss despite limited efficacy; a notable case achieved 36-month survival post-infusion [145]. To improve outcomes, a novel EGFRvIII CAR equipped with T-cell-engaging antibody molecules (TEAMs) [146] was infused intraventricularly (INCIPIENT trial, NCT05660369), resulting in rapid tumor regression in all three treated patients, albeit with transient effects in two and no grade ≥ 3 toxicity [147].

EphA2, overexpressed in ~60–90% of GBMs, was targeted in a small intravenous Phase I trial (NCT02575261). Among the three patients, two developed grade 2 cytokine release syndrome (one with pulmonary edema); clinical outcomes included one stable disease and two progressions, with survival ranging from 86 to 181 days. This trial has now been withdrawn.

HER2-targeted CAR T-cells have similarly been administered via intrathecal/intraventricular routes in adults and young adults (NCT03500991), with manageable safety profiles, side effects included headaches and neurological symptoms, but efficacy data remain limited [148].

## 21. Integrating Artificial Intelligence for Precision CAR T-Cell Therapy in Solid Tumors

Artificial intelligence (AI) technologies are accelerating the development of CAR and TCR therapies. Emerging deep learning (DL) and machine learning (ML) models enable the design of optimized CARs targeting multiple tumor antigens and improve the prediction of CAR binding affinity, while natural language processing (NLP) tools mine the biomedical literature and clinical databases for insights [149]. Computer vision supports real-time visualization of CAR T-cell morphology during manufacturing, and Reinforcement Learning is being explored to fine-tune dosing regimens, together improving precision across the CAR T pipeline [150].

AI models can prioritize optimal CAR targets by analyzing multi-omics datasets, including RNA sequencing, proteomics, and spatial transcriptomics, to identify actionable antigens with high tumor specificity and minimal expression in normal tissues [151]. Additionally, AI is being employed to predict antigen escape, T-cell exhaustion, and immunosuppressive features of the TME, enabling the rational design of CAR constructs capable of resisting or even reprogramming the hostile TME [152].

Beyond target identification, AI-driven models are advancing the design of CARs themselves. Generative design tools now assist in optimizing the single-chain variable fragment (scFv), intracellular signaling domains, and hinge/spacer regions to enhance tumor penetration, persistence, and activation profiles [150].

Automated manufacturing platforms, informed by sensor and imaging data, enhance CAR T product consistency and reduce batch failures, making therapies more scalable and cost-effective. These AI-driven tools hold promise for improving patient selection, standardizing quality control, and streamlining regulatory processes [153].

Despite these opportunities, significant barriers remain. The field faces challenges including limited and biased training datasets, lack of reproducible external validation, and “black-box” algorithm opacity that undermines clinician trust.

## 22. Barriers to CAR T-Cell Therapy in Solid Tumors

CAR T-cell therapy encounters substantial challenges in the treatment of solid tumors, which are distinct from those observed in hematological malignancies. A major barrier is tumor heterogeneity, encompassing variability in antigen expression and intrinsic genetic mutations, which frequently results in antigen escape and incomplete tumor regression. The immunosuppressive TME, composed of regulatory T-cells, myeloid-derived suppressor cells, tumor-associated macrophages, and immunosuppressive cytokines such as transforming growth factor-beta (TGF-β) and interleukin-10 (IL-10), further undermines CAR T-cell efficacy by inhibiting T-cell activity and facilitating immune evasion. Additionally, structural barriers, including a dense extracellular matrix and abnormal tumor vasculature, restrict CAR T-cell infiltration and persistence, while metabolic constraints, such as glucose depletion and hypoxia, exacerbate functional impairment. Collectively, these challenges underscore the necessity for innovative strategies to enhance the therapeutic efficacy of CAR T-cell therapy in solid tumors, as outlined in the subsequent subsections.

### 22.1. Tumor Heterogeneity

Solid tumors are characterized by significant biological heterogeneity.

Antigen Heterogeneity: Antigen heterogeneity is a significant challenge when targeting solid tumors with CAR T-cell therapies. Solid tumors often express a diverse array of antigens, and the levels of these antigens can vary within a single tumor or among different tumors in the same patient [154].

Tumor-Intrinsic Heterogeneity: Each tumor may have distinct genetic mutations leading to the expression of various neoantigens. This intra-tumoral heterogeneity poses challenges for CAR T-cells trained to target specific antigens, as a subpopulation of tumor cells may exist that does not express the targeted antigen. Consequently, this antigen escape can lead to incomplete tumor regression and treatment failure [155].

### 22.2. Tumor Microenvironment’s Role

The TME can further complicate antigen expression. For example, hypoxic conditions can downregulate the expression of certain surface antigens, rendering them less visible to CAR T-cells [156]. Moreover, the selection pressure of CAR T-cell therapy may favor tumor cells that can evade recognition, promoting the survival of antigen-negative cells.

Immunosuppressive TME: The TME in solid cancers is a critical factor that significantly hinders CAR T-cell efficacy. Unlike hematological malignancies, where cancer cells are dispersed in the bloodstream, solid tumors are embedded within a robust and complex microenvironment that includes stromal cells, extracellular matrix (ECM), regulatory T-cells (Tregs) and other immune cells, and various cytokines and chemokines. Additionally, TME-derived metabolites such as kynurenine and lactate can suppress effector T and NK cells, drive Treg expansion, and skew macrophages toward an immunosuppressive phenotype. Tumor-derived extracellular vesicles may further contribute to CAR T dysfunction. These cellular and metabolic barriers highlight the complexity of the TME and underscore the need for combinatorial strategies to enhance CAR T efficacy [157].

Immunosuppressive Cell Populations: Within the TME, various cell types such as Tregs, myeloid-derived suppressor cells (MDSCs), and tumor-associated macrophages (TAMs) often function to suppress T-cell activity. Tregs can inhibit effector T-cells, while MDSCs can create a hostile environment through the secretion of immunosuppressive factors like arginase and nitric oxide [158]. This immune suppression can dampen the efficacy of CAR T-cells, limiting their ability to attack tumor cells.

Cytokine Production: The TME is frequently rich in immunosuppressive cytokines, including transforming growth factor-beta (TGF-β) and interleukin-10 (IL-10). These cytokines not only contribute to immune evasion but also can directly inhibit the proliferation and function of CAR T-cells [159]. Moreover, the presence of pro-inflammatory cytokines like IL-6 can lead to cytokine release syndrome, complicating treatment further.

Metabolic Conditions: A key metabolic hallmark of both tumor and effector T-cells is aerobic glycolysis, known as the Warburg effect, which creates a competition for glucose in nutrient-deprived regions of the tumor, impairing T-cell expansion and function [160]. This competition is compounded by the accumulation of lactic acid and other metabolites, such as those from glutaminolysis, which lower pH and suppress immune cell activity, including CAR T-cells. Hypoxia, due to poor vascularization, further promotes tumor growth and immune evasion, partly by stabilizing HIF-1α in TAMs, leading to the secretion of matrix-degrading enzymes and pro-tumor factors. Moreover, the depletion of amino acids like L-arginine and tryptophan, driven by overactive enzymes such as IDO and TDO, results in T-cell dysfunction and undermines anti-tumor immunity [161].

### 22.3. Physical Barriers

Stromal Components: The ECM forms a physical barrier that can limit CAR T-cell infiltration into tumor tissues. In solid tumors, a dense stromal compartment may impede the movement of immune cells, preventing adequate interaction between CAR T-cells and target tumor cells. The physical nature of the ECM can also affect the trafficking and persistence of CAR T-cells, ultimately influencing therapeutic outcomes [162].

Tumor Vasculature: The abnormal blood vessel architecture in solid tumors often results in imperfect perfusion and nutrient supply, leading to areas of hypoxia within the tumor. The impaired vascular network can further reduce the trafficking of CAR T-cells into the tumor [163].

### 22.4. Antigen Escape

Antigen escape remains a critical obstacle limiting the efficacy and durability of CAR T-cell therapy. Traditional CARs are engineered to target a single surface molecule (e.g., CD19), which works well when all tumor cells express this antigen. However, tumor heterogeneity often leads to the emergence of antigen-low or antigen-negative clones, resulting in eventual disease relapse [164]. To combat this, researchers are developing several innovative approaches:

Multi targeted CARs—These include bispecific or tandem CAR (TanCAR) constructs that recognize multiple antigens, reducing the likelihood of tumor cells escaping detection [52].

Enhanced CAR sensitivity—Optimizing affinity and activation thresholds to detect and eliminate cells with low antigen expression [165].

Safety mechanisms for broader targeting—Refining designs to safely target shared tumor-associated antigens while minimizing off-tumor toxicity [164].

Immune engagement strategies—Engineering CAR T-cells to stimulate endogenous immune responses, promoting broader epitope spreading and reducing reliance on a single antigen [166].

These multi-pronged strategies aim to substantially boost CAR T-cell efficacy, prolong remissions, and improve cure rates.

### 22.5. “On-Target Off-Tumor” Toxicity in CAR T-Cell Therapy

“On-target off-tumor” toxicity occurs when CAR T-cells recognize and attack healthy tissues expressing the target antigen, leading to severe adverse effects. This remains a major challenge in CAR T-cell therapy, particularly for solid tumors where TAAs are often shared with normal cells.

Key Challenges include a lack of truly TAA, high-affinity CARs and tonic signaling. Many TAAs (e.g., HER2, EGFR, MUC1) are expressed at low levels on healthy tissues leading to potential for toxicity in those cells. High affinity CARs have an excessive binding affinity which can lead to activation against low-antigen-density normal cells. Tonic signaling in the form of persistent CAR activation in the absence of antigens can cause exhaustion and unintended cytotoxicity.

Several strategies have been evaluated to mitigate “on-target off-tumor” toxicity.

- Affinity Tuning
  Lowering the binding affinity of CARs can enhance selectivity for tumor cells overexpressing the antigen while sparing normal cells with low antigen density. A study by Liu et al. (2022) demonstrated that reducing HER2-CAR affinity prevented off-tumor toxicity while maintaining anti-tumor efficacy [167].
- Humanized or Fully Human scFvs
  Murine-derived single-chain variable fragments (scFvs) can induce immunogenicity. Humanized or fully human CAR designs reduce immunogenicity and improve safety. As an example, a fully human CD19 CAR T-cell (lisocabtagene maraleucel) showed reduced immunogenicity compared to murine-based constructs [168].
- Logic-Gated CARs (AND, OR, NOT Gates)
  These CARs require multiple antigens for activation (AND gate) or inhibit activity in the presence of a normal tissue marker (NOT gate). As far back as 2013, a PSCA + PSMA AND-gated CAR T-cell demonstrated enhanced tumor specificity in prostate models [169].
- Transient CAR Expression (mRNA CARs, Switchable CARs)
  Short-lived CAR T-cells (via mRNA electroporation) or switchable CARs (controlled by an external antibody) can limit prolonged activity and mitigate toxicity. Zhao et al. demonstrated that mRNA-based CAR T-cells targeting mesothelin showed transient activity, thereby reducing toxicity [170].
- Inhibitory CARs (iCARs)
  iCARs co-express an inhibitory receptor (e.g., PD-1, CTLA-4) that suppresses CAR-T activity upon binding to normal tissue markers. This approach has been validated preclinically to prevent off-tumor toxicity, for example, in therapies targeting antigens with restricted expression on healthy tissues [171].
  Overall, mitigating “on-target off-tumor” toxicity requires a combination of affinity optimization, humanized designs, logic-gated systems, transient expression, and inhibitory mechanisms. Recent advances in synthetic biology and protein engineering are improving the safety profile of CAR T-cell therapies, particularly for solid tumors.

## 23. Development Strategies for Enhancing CAR T-Cell Therapy in Solid Cancers

Several innovative strategies are being explored to enhance CAR T-cell efficacy and overcome limitations in solid cancers. These strategies include combining CAR T-cell therapy with immune checkpoint inhibitors (ICIs), utilizing CRISPR-Cas9 gene editing to optimize CAR T-cell function, and other advanced approaches such as “armored” CAR T-cells, bispecific CAR T-cells, and localized delivery methods.

### 23.1. Combining CAR T-Cell Therapy with Immune Checkpoint Inhibitors

One of the most promising strategies to enhance CAR T-cell therapy is its combination with immune checkpoint inhibitors. Immune checkpoints, such as PD-1, PD-L1, and CTLA-4, are critical regulators of the immune response, helping to prevent autoimmunity by limiting T-cell activation. However, tumors often hijack these immune checkpoints to evade immune surveillance. By inhibiting checkpoint pathways, immune checkpoint inhibitors can reinvigorate T-cell responses against tumor cells [172].

The combination of CAR T-cells and ICIs can enhance both the expansion and persistence of CAR T-cells within the TME. ICIs such as anti-PD-1 block the PD-1/PD-L1 interaction, which normally suppresses T-cell activity. By removing this inhibitory signal, ICIs can increase the activation and proliferation of CAR T-cells, enabling them to better target and destroy cancer cells [173].

In a study, Srivastava et al. demonstrated that combining immunogenic chemotherapy with CAR T-cell therapy and PD-L1 blockade significantly enhances tumor targeting and control in lung cancer models. In Kras^LSL G12D/+; p53^f/f mice engineered to express the ROR1 antigen, a relevant model for NSCLC and TNBC, CAR T-cells alone delivered after standard lymphodepletion (cyclophosphamide and fludarabine) showed poor tumor infiltration, rapid dysfunction, and only transient anti-tumor activity similar to what has been observed in clinical settings [174]. However, when oxaliplatin was added to the lymphodepletion regimen, it triggered immunogenic cell death and activated tumor-resident macrophages to secrete chemokines (e.g., CXCL9/10, CCL5). This led to improved CAR T-cell infiltration, activation, and tumor remodeling; while CAR T-cells and oxaliplatin alone modestly controlled tumor growth, full tumor regression and enhanced survival required adding PD L1 blockade [174].

In addition to direct effects on CAR T-cells, ICIs may alter the TME to make it more permissive for CAR T-cell activity. For example, ICIs can reduce the numbers of immune suppressor cells (such as regulatory T-cells and myeloid-derived suppressor cells) within the tumor, further enhancing the anti-tumor immune response [175]. Moreover, combination therapy can potentially overcome resistance mechanisms that may arise when CAR T-cells alone are insufficient to fully eradicate the tumor.

Two early-phase clinical trials have evaluated the combination of CAR T-cell therapy with PD-1/PD-L1 checkpoint blockade. In a study by Chong et al., a patient with refractory diffuse large B-cell lymphoma received CD19-targeted CAR T-cells following lymphodepleting chemotherapy. When disease progression and elevated PD-L1 expression were noted, pembrolizumab was administered starting on day 26. This intervention led to a decrease in PD-1/EOMES co-expressing T-cells, expansion of CAR T and TCRβ clones, and tumor regression by day 45 [176]. In a separate trial, Heczey et al. treated 11 patients with relapsed/refractory neuroblastoma using various combinations of CAR T-cells, lymphodepletion, and pembrolizumab. While the regimens were well tolerated and lymphodepletion improved CAR T expansion, pembrolizumab did not significantly enhance persistence, though one complete response was noted [177]. Despite these early signals of efficacy, challenges remain, including the need for repeated antibody dosing, limited tumor penetration, and systemic toxicity. Genetically engineering CAR T-cells for intrinsic checkpoint resistance may offer a more durable and targeted solution.

### 23.2. Combination with Radiotherapy

Radiotherapy, beyond its well-established role in directly eradicating tumor cells, also exerts profound immunomodulatory effects within the TME. It stimulates immune activation by releasing damage-associated molecular patterns (DAMPs) and tumor-associated antigens, thereby enhancing antigen presentation [178]. This cascade activates dendritic cells and antigen-presenting cells through upregulation of MHC molecules and the secretion of immunogenic signals such as calreticulin, HMGB1, and ATP [178]. These changes help initiate and amplify endogenous anti-tumor immunity, which can synergize with the activity of adoptively transferred CAR T-cells. Moreover, radiotherapy can reprogram the TME by decreasing immunosuppressive populations like MDSCs and M2 macrophages, while favoring M1-like macrophage phenotypes that support anti-tumor responses [178].

Additionally, radiotherapy promotes CAR T-cell trafficking and infiltration into tumors by inducing chemokine production (e.g., CXCL9-11) and increasing adhesion molecule expression (e.g., ICAM-1, VCAM-1) on vascular endothelial cells. These changes facilitate CAR T-cell homing and transendothelial migration. Radiotherapy also improves tumor vascular normalization and oxygenation, mitigating hypoxia-related resistance and supporting CAR T-cell expansion at the tumor site. Importantly, while radiotherapy may induce radioresistance in some tumor cells, these resistant cells often remain vulnerable to T-cell-mediated attack [179]. Therefore, the integration of radiotherapy with CAR T-cell immunotherapy represents a promising strategy to enhance tumor control through complementary mechanisms.

A Phase II clinical trial evaluating CAR T-therapy in relapsed/refractory diffuse large B-cell lymphoma (R/R DLBCL) demonstrated that patients who received bridging radiotherapy (2 Gy × 20 fractions) prior to CAR T infusion experienced a higher overall response rate and reduced toxicity compared to those given bridging chemotherapy. The study utilized CAR T-cells targeting CD19, CD20, or CD22, with dual-antigen targeting based on relapsed antigen expression. Notably, radiotherapy appeared to mitigate the severity of cytokine release syndrome and neurotoxicity, suggesting that it is a safe and effective strategy for tumor burden reduction before CAR T-cell administration in high-risk DLBCL patients [180].

#### Unique Challenges of “Homing” in CAR T-Cell Therapy with Radiotherapy

The concept of homing in CAR T-cell therapy, particularly in the context of radiotherapy, represents a significant challenge. Traditionally associated with chemokine receptor engineering to enhance tumor infiltration [148], homing enables CAR T-cells to migrate efficiently to tumor sites. Although radiotherapy can improve antigen presentation, it simultaneously disrupts homing by inducing inflammation that recruits immunosuppressive cells such as myeloid-derived suppressor cells (MDSCs) and regulatory T-cells (Tregs) [158,175], while also causing vascular damage and fibrosis that impair perfusion [163]. Furthermore, temporal and spatial radiation effects—such as uneven chemokine gradients, antigen downregulation [145,180], and especially variable infusion timings [179], further complicate homing. To address these barriers, potential strategies include synchronizing CAR T-cell infusion with peak chemokine expression, engineering dual chemokine receptors, and combining therapy with immune checkpoint inhibitors [172,176] to mitigate TME alterations.

### 23.3. CRISPR-Cas9 Gene Editing to Optimize CAR T-Cell Efficacy and Safety

CRISPR-Cas9 is a revolutionary gene-editing technology that allows precise modification of the genome, enabling the addition, deletion, or alteration of specific genes. In the context of CAR T-cell therapy, CRISPR-Cas9 gene editing enables precise alteration of CAR T-cells to boost tumor specificity, persistence, and resistance to adverse microenvironments [181]. Unlike conventional viral-based CAR integration methods, CRISPR allows for precise insertion of CAR constructs into defined genomic loci, most notably the T-cell receptor alpha constant (TRAC) locus, leading to uniform CAR expression and reduced tonic signaling, which enhances CAR T-cell persistence and function [182]. Furthermore, by knocking out genes encoding endogenous T-cell receptors and HLA molecules, CRISPR facilitates the creation of universal, allogeneic CAR T-cells, eliminating the need for autologous cell harvesting and reducing manufacturing time and costs [183].

Beyond basic editing, CRISPR enables multiplex gene disruption to overcome intrinsic and extrinsic barriers in TME. For example, co-disruption of PD-1, LAG3, or CTLA-4 attenuates inhibitory checkpoint signaling and enhances effector function. Simultaneous targeting of exhaustion-associated transcription factors (e.g., TOX, NR4A) can reprogram T-cells toward a memory-like phenotype with superior in vivo persistence. Additionally, gene editing of metabolic regulators and cytokine signaling pathways (e.g., knocking out TGF-β receptor II or SOCS1) has shown promise in preclinical models to mitigate TME-driven suppression and augment tumor infiltration and cytotoxicity [182].

Despite its promise, CRISPR-based editing introduces challenges. Concerns over off-target effects, large chromosomal rearrangements, and p53 pathway activation necessitate stringent quality control during clinical manufacturing. Moreover, the immunogenicity of the bacterial Cas9 protein could impair persistence of engineered cells in vivo. Nevertheless, early-phase clinical trials have demonstrated the feasibility and preliminary safety of multiplex-edited CAR or TCR-engineered T-cells [181]. As gene-editing technologies continue to evolve, through base editing, prime editing, or Cas variants with reduced immunogenicity, the integration of CRISPR/Cas9 into CAR T-cell therapy holds significant potential to reshape personalized and off-the-shelf cellular immunotherapy.

### 23.4. Other Development Strategies

In addition to combining CAR T-therapy with immune checkpoint inhibitors and gene editing, other strategies are being explored to enhance CAR T-cell functionality in solid tumors.

#### 23.4.1. Armored CAR T-Cells

“Armored” CAR T-cells are engineered to resistant the immunosuppressive forces present within the TME. For example, researchers are adding cytokines such as IL-12 or IL-15 to CAR T-cells to enhance their persistence and anti-tumor activity. IL-12, a potent pro-inflammatory cytokine, can promote T-cell activation, increase the cytotoxic activity of CAR T-cells, recruit myeloid and NK cells, and help overcome the suppressive effects of regulatory T-cells and myeloid-derived suppressor cells [67]. Several clinical trials are underway to assess the safety and efficacy of armored CAR T-cells in solid tumors, and early results suggest that this strategy may improve tumor infiltration and response rates. In glioblastoma models, CAR T-cells engineered to co-express inducible IL 12 and IL 18 upon tumor engagement have demonstrated profound pro-inflammatory remodeling of the TME, resulting in superior tumor eradication compared to single-cytokine designs. Shih et al. reported that these dual-armored CAR T-cells (IL 12.DR 18) enhanced NK and macrophage activation but also generated transient toxicity; toxicity was effectively reduced by pooling with CAR T-cells also secreting anti VEGF scFv, maintaining anti-tumor efficacy [184].

CAR T-cells expressing 4-1BB ligand (4-1BBL) provide autocrine and paracrine costimulatory signals that augment T-cell persistence and resistance to exhaustion. Additionally, PD-1 checkpoint blockade, either via CRISPR-mediated disruption or secretion of anti–PD-1 scFv, enables armored CAR T-cells to maintain effector function within checkpoint-enriched solid tumors [67,185].

Armored CAR T-cells also show potential as a salvage treatment in patients progressing following prior CAR T-cell therapy. A recent Phase 1 trial by Svoboda et al. using huCART19-IL18 (IL-18–armored, humanized CD19 CAR T) in 21 lymphoma patients relapsing after prior CD19 CAR T showed ORR 81% and CR 52% at 3 months, with durable remissions (median DOR 9.6 months). Toxicity was manageable (CRS mostly grade 1–2; ICANS mild, no fatal events). Rapid 3-day manufacturing proved feasible. These findings support huCART19-IL18 as a promising salvage CAR T strategy [186].

There is also interest in using dual armored CAR T-cells for pan-solid tumor usage, with an ongoing trial (NCT06198296) recruiting at the Baylor College of Medicine using IL-15 and IL-12 armored GPC3-CAR T-cells to be given in all Glypican-3 (GPC3) positive solid tumors [187].

#### 23.4.2. Bispecific and Multi-Specific CAR T-Cells

Bispecific CAR T-cells are designed to target two different tumor-associated antigens simultaneously. This approach is particularly beneficial for solid tumors, which often display heterogeneous antigen expression. By targeting multiple antigens, bispecific CAR T-cells can reduce the risk of antigen escape and improve tumor cell targeting. A few platforms, including TanCAR and dual CAR platforms, are currently in development [188].

An example is engineered T-cells that co-express a GPC2-directed CAR and secrete a bispecific innate immune cell engager (BiCE) targeting both GD2 and CD16a, enhancing engagement of innate effectors in neuroblastoma models. These bicistronic GPC2.CAR-GD2.BiCE T-cells demonstrated robust cytotoxicity against GPC2-positive cells in vitro, while simultaneously promoting activation of NK cells and macrophages via secreted GD2.BiCE, effectively redirecting innate immunity toward GD2-expressing tumors. In patient-derived xenograft-bearing mice reconstituted with human CD16a+ innate cells, this hybrid platform improved intra-tumoral retention of NK cells and achieved greater tumor suppression compared to conventional CAR T-therapy, addressing limitations posed by antigen heterogeneity and escape [189].

Several clinical trials are investigating the safety and efficacy of bispecific CAR T-cell therapies for different cancers, including a HER2/PD-L1 dual-targeting CAR T-cells in patients with HER2-positive solid tumors (NCT04684459). These studies suggest that bispecific CAR T-cells may provide a more effective treatment option, particularly for tumors that express multiple antigens or those that are prone to antigen loss.

### 23.5. Multi-Antigen CAR T-Cell Strategies

The recognition that single-targeted CAR T-cells are inadequate to address the complexity of solid tumors has highlighted the importance of multi-antigen approaches. Solid tumors display substantial antigen heterogeneity and employ escape mechanisms that render monovalent CAR T-cell therapies vulnerable to treatment failure [154,164]. Multi-antigen CAR designs, such as bispecific or tandem CAR (TanCAR) constructs, enable simultaneous targeting of multiple tumor-associated antigens (TAAs), thereby reducing the risk of antigen-negative tumor cell survival [52]. By engaging diverse antigenic profiles within a single tumor or across metastatic sites, these strategies hold promise for improving both specificity and therapeutic efficacy.

A particularly promising innovation is the application of logic gating, pioneered by Cole Roybal and Wendell Lim, which incorporates synthetic biology principles into CAR T-cell design. Logic-gated CARs, such as AND-gate and NOT-gate systems, employ Boolean logic to regulate T-cell activation based on the co-expression of multiple antigens. For example, an AND-gate CAR requires the simultaneous presence of two specific antigens (e.g., HER2 and MUC1) to induce cytotoxicity, thereby minimizing off-target effects in healthy tissues expressing only one antigen [190]. In contrast, NOT-gate CARs suppress activation when an inhibitory antigen—such as a normal tissue marker—is detected, thus enhancing safety [191]. This level of precision is especially critical in solid tumors, where on-target, off-tumor toxicity remains a major concern [164].

Complementing these efforts, the optimization of costimulatory domains has emerged as a critical area of innovation. Goodman et al. (2022) in Science Translational Medicine demonstrated that tailoring costimulatory domains—such as CD28, 4-1BB, or OX40—can substantially enhance CAR T-cell persistence and effector function in solid tumor models. Their findings indicate that combining 4-1BB with novel domains like ICOS improves metabolic fitness and resistance to the immunosuppressive TME, whereas CD28 variants promote early activation but may increase susceptibility to exhaustion [192]. Current research is investigating synergistic domain pairings and their effects on T-cell longevity, aiming to achieve an optimal balance between activation and exhaustion within the hostile TME of solid tumors [159].

These multi-antigen strategies, combined with logic gating and optimized costimulatory domains, represent a transformative step toward personalized and more robust CAR T-cell therapies. Future directions may include the integration of multi-omics data to identify optimal antigen combinations and the refinement of domain engineering to adapt CAR T-cells to the dynamic conditions of the tumor microenvironment, reflecting recent advancements in the field.

Another promising development is the localized delivery of CAR T-cells directly to the tumor site. This approach can help minimize systemic side effects and enhance the concentration of CAR T-cells at the tumor site. Localized delivery is being explored through techniques such as intra-tumoral injections or the use of microcapsules that release CAR T-cells in a controlled manner. These methods could offer a more effective way of overcoming the challenges of immunosuppressive TME, which often limits the effectiveness of systemic CAR T-cell therapy.

One of the principal challenges impeding the efficacy of CAR T-cell therapy in solid tumors lies in the suboptimal trafficking and infiltration of CAR T-cells into the TME, often compounded by systemic toxicities and immunosuppressive barriers. Locoregional delivery has emerged as a promising strategy to circumvent these limitations by enabling higher local concentrations of effector cells while mitigating systemic exposure.

Various locoregional routes have been explored depending on tumor location and accessibility. Intra-arterial infusion, particularly hepatic artery infusion (HAI), has shown promise in hepatic malignancies and pancreatic cancer, with clinical studies reporting reduced systemic toxicities and improved CAR T-cell persistence [189]. Trials such as HITM-SIR (NCT02416466) and HITM-SURE (NCT02850536) demonstrated favorable safety profiles and prolonged survival in patients with metastatic colorectal and pancreatic cancer, notably achieving complete metabolic responses in some cases using pressure-enabled HAI systems. Similarly, image-guided intra-tumoral administration allows precise delivery into tumor lesions, facilitating local immune activation and leveraging abscopal effects. Early-phase trials (e.g., NCT01818323) have demonstrated disease stabilization and prolonged CAR T-cell persistence in head and neck squamous carcinoma.

Cavitary delivery routes, including intraperitoneal, intrapleural, and intraventricular infusions, have been particularly impactful in tumors confined to anatomical cavities. Intrapleural administration of mesothelin-targeted CAR T-cells in malignant mesothelioma has shown durable systemic responses and was further potentiated by anti-PD-1 checkpoint blockade (NCT02414269). In the CNS, circumventing the blood–brain barrier via intraventricular or intracavitary routes (e.g., Ommaya reservoir) has allowed for effective treatment of glioblastoma and leptomeningeal disease with IL-13Rα2 or B7-H3-targeted CAR T-cells, with some studies reporting complete lesion clearance and prolonged remission without grade ≥ 3 neurotoxicity (NCT02208362, NCT03500991). These findings support the evolving paradigm in which locoregional CAR T-cell delivery not only addresses physical barriers of solid tumor targeting but may also enable synergy with endogenous and combinatorial immunotherapies [193].

In addition to physical locoregional delivery routes, innovative nanomedicine strategies are emerging to streamline CAR T-therapy by enabling in situ gene delivery, obviating the need for ex vivo manufacturing. Fan et al. reviewed the use of biocompatible lipid or polymeric nanoparticles for targeted delivery of CAR-encoding mRNA or DNA directly to T-cells within the patient, establishing a “universal” CAR T-platform that promises reduced cost, faster deployment, and simpler logistics compared to personalized ex vivo approaches [194]. These systems exploit selective organ targeting (SORT) lipid nanoparticles to achieve T-cell transfection and CAR expression in vivo, demonstrating effective anti-tumor responses in preclinical models. Nanocarriers can also co-deliver immunomodulatory agents or TME-modulating payloads, offering the potential to integrate locoregional targeting with immune activation and safety control. While clinical translation remains forthcoming, this nanoparticle-mediated strategy exemplifies a future in which CAR T delivery converges with gene therapy and nanotechnology, expanding the therapeutic toolkit for solid tumors.

Injectable Hydrogel Niches: Grosskopf et al. have proposed an injectable hydrogel method for CAR T-cell delivery, aiming to create a localized inflammatory niche to improve T-cell infiltration and persistence [195]. This hydrogel acts as a temporary biocompatible depot releasing cytokines that may attract and activate CAR T-cells.

## 24. Quality-of-Life Considerations, Real-World Implementation Challenges Including Costs, Accessibility, and Scalability

In general, the quality of life in patients receiving CAR T-cell therapy is well maintained with most patients reporting an improvement after an early dip during the first 1–3 months during which most of the common adverse events are seen. Tolerability across patient populations is seen as well, including the young as well as the old and frail. A common comparator of this new treatment paradigm has been with traditional stem cell transplants and it has been seen that any decline in QoL and its recovery has been seen to be better with CAR T-cell therapy as compared to both autologous and allogenic stem cell transplants [196]. As this is still an emerging technology, further data is needed to study its impact in various tumor types across geographical and racial variations. Real-world CAR T implementation is limited by **high upfront costs**, complex **manufacturing and supply chain logistics**, and the need for **specialized centers with intensive monitoring capacity.** Barriers include **geographic inaccessibility, insurance disparities, and workforce training gaps,** raising equity concerns and slowing broad scalability. To this date, there are only a dozen approved CAR T-cell products worldwide mainly for three hematological malignancies, namely, B-ALL, B-NHL and Multiple Myeloma. The key bottlenecks in further development include identification of an ideal target, transduction (manufacture of the viral vector), developing new models of CARs with higher efficacy and lower potential adverse events, and to bring down costs. CAR T-cell therapy has been accessible to developing countries at lower cost only for the past two years after indigenous CAR T products were developed by countries such as India and China. Scalability in these regions involves significant public–private partnerships and investor-funded initiatives which have helped greatly to bring down the prices of current CAR T-cell therapy to 1/10th the original cost of the innovator with comparable efficacy and side effect profiles [197]. The same challenges exist for CAR T-cells for solid malignancies albeit at a higher scale, as the targets necessary for efficient treatment of solid tumors necessitate integration of high-end technologies including gene editing with CRISPR/Cas9 as mentioned above, improved CAR T-molecular structure with possible need for multiple target recognition, better costimulatory molecules, more precise methods of cell kill without collateral damage, and integration with other forms of therapy such as checkpoint inhibitors or cytokines.

## 25. Conclusions

CAR T-cell therapy has transformed the treatment paradigm for hematological malignancies, offering durable responses in otherwise refractory disease. However, translating this success to solid tumors remains challenging due to complex barriers such as the immunosuppressive TME, antigen heterogeneity, physical barriers to trafficking, and on-target/off-tumor toxicity. Despite these hurdles, recent clinical progress—including the FDA approval of afami-cel in synovial sarcoma and lifileucel in melanoma—demonstrates that genetically engineered T-cell therapies can be feasible and effective in select solid tumors.

Across a wide range of malignancies, including lung, colorectal, breast, ovarian, pancreatic, and glioblastoma, clinical trials are actively testing advanced CAR designs such as dual-targeted CARs, armored constructs, regional delivery, and allogeneic platforms. The most promising strategies appear to involve rational antigen selection, modulation of the TME, and combination approaches with checkpoint inhibitors or cytokines.

Looking forward, greater understanding of CAR T-cell kinetics, resistance mechanisms, and predictive biomarkers will be essential to refining patient selection and improving efficacy. Real-world implementation will also require addressing scalability, cost, and toxicity management. While still early in development, CAR T-cell therapy in solid tumors is steadily evolving, and with continued innovation, it has the potential to offer meaningful clinical benefit across a broader range of solid cancers.

## Figures and Tables

**Table 1:** Signaling differences between native T-cells and CAR T-cells.

Signaling Component	Native T-Cell Function	CAR T-Cell Equivalent	Purpose
Signal 1 (Recognition)	TCR + CD3 complex sees peptide/MHC	scFv domain binds surface antigen	Target recognition and initial activation trigger
Signal 2 (Costimulation)	CD28 binds B7 on an APC	Costimulatory Domain (e.g., CD28, 4-1BB)	Full, robust activation; prevents anergy; promotes expansion and persistence
Activation Signal	ITAMs on CD3ζ chain transmit signal	ITAMs on CD3ζ domain transmit signal	Initiating the downstream signaling cascade for T-cell functions (killing, cytokine release)

**Table 2:** Completed and ongoing CAR T-cell therapy clinical trials recruiting triple-negative breast cancer patients. Table as of 19 August 2025.

NCT Number	Study Status	Study Title	Interventions	Design	Sponsor Country and Institution	Key Results
NCT05341492	Recruiting (ongoing)	A Single-arm, Open, Exploratory Clinical Study Evaluating the Safety and Efficacy of EGFR/B7H3 CAR-T in Patients With EGFR/B7H3-positive Advanced Solid Tumors (Lung and Triple-negative Breast Cancer)	Biological: EGFR/B7H3 CAR-T	Early Phase 1, single arm, ~30 patients	Second Affiliated Hospital of Guangzhou Medical University, China	No formal outcome data yet available
NCT01837602	Completed	Clinical Trial of Autologous cMet Redirected T Cells Administered Intratumorally in Patients With Breast Cancer	Biological: cMet RNA CAR T cells	Phase 1, interventional, ~6 evaluable patients	University of Pennsylvania, United States	Safe (≤Grade 1 toxicity); CAR T mRNA detected in blood/tumor; tumor necrosis and inflammatory response observed
NCT02580747	start ~October 2015, possibly ended ~November 2017; current status unknown	Clinical Study of Chimeric Mesothelin Antigen Receptor-modified T Cells in Relapsed and/or Chemotherapy Refractory Malignancies	Biological: anti-meso-CAR vector transduced T cells	Phase 1, interventional, ~20 participants	Chinese PLA General Hospital, China	No outcomes publicly available as of now
NCT06682793	Recruiting	A Seamless Phase 1/2 Study to Evaluate the Safety and Efficacy of A2B395, an Allogeneic Logic-gated Tmod™ CAR T, in Heterozygous HLA-A*02 Adults With Recurrent Unresectable, Locally Advanced, or Metastatic Solid Tumors That Express EGFR and Have Lost HLA-A*02 Expression	Biological: A2B395 Diagnostic Test: xT CDx with HLA-LOH assay	Seamless Phase 1/2; open-label, interventional, multi-center, ~240 participants	A2 Biotherapeutics, Inc., United States	The key milestone for this trial is that the first patient has been dosed (June 2025). No clinical outcome data (safety/efficacy) are available yet.
NCT04107142	Not yet recruiting	A Phase I Dose-escalation Trial to Evaluate Haploidentical/Allogeneic Natural Killer Group 2D Ligand (NKG2DL)-Targeting Chimeric Antigen Receptor-grafted Gamma Delta (γδ) T Cells (CTM-N2D) in Subjects With Relapsed or Refractory Solid Tumour	Biological: Adoptive Cell Transfer of NKG2DL-targetting Chimeric Antigen Receptor-grafted Gamma Delta T cell	Open label, single-center, dose-escalation, Phase I study, ~10 participants	CytoMed Therapeutics Pte Ltd., Singapore	Results pending
NCT02587689	Recruiting	Phase I/II Study of Anti-MUC1 CAR T Cells for Patients With MUC1+ Advanced Refractory Solid Tumor	Biological: anti-MUC1 CAR T Cells	Interventional, Phase 1/2, single-arm, open label, ~20 participants	PersonGen BioTherapeutics (Suzhou) Co., Ltd., China	Results pending
NCT02706392	Terminated due to slow accruals.	Phase I Study of Adoptive Immunotherapy for Advanced ROR1+ Malignancies With Defined Subsets of Autologous T Cells Engineered to Express a ROR1-Specific Chimeric Antigen Receptor	Other: Laboratory Biomarker Analysis Biological: ROR1 CAR-specific Autologous T-Lymphocytes	Phase I, interventional, single-arm, open label, ~21 participants	Fred Hutchinson Cancer Center, United States	Safety Profile: No dose-limiting toxicities were observed. Efficacy Observations: In a subset of patients, evidence of CAR-T cell expansion and potential anti-tumor activity was noted.
NCT06347068	Recruiting	Study of Administration of T Cells Expressing B7-H3 Specific Chimeric Antigen Receptors and Containing the Inducible Caspase 9 Safety Switch in Subjects With Triple Negative Breast Cancer	Biological: iC9-CAR.B7-H3 T Cell Therapy Drug: cyclophosphamide Drug: fludarabine	Interventional, Phase 1, single arm, open label, ~42 participants	UNC Lineberger Comprehensive Cancer Center, United States	Results pending
NCT02792114	The trial is actively ongoing but not recruiting new participants.	A Phase I Clinical Trial to Evaluate the Safety and Tolerability of Mesothelin-Specific Chimeric Antigen Receptor-Positive T Cells in Patients With Metastatic Mesothelin-Expressing Breast Cancer	Drug: Cyclophosphamide Biological: Mesothelin-targeted T cells Drug: AP1903	Interventional, Phase I, single arm, open label, ~186 participants	Memorial Sloan Kettering Cancer Center, United States	Results pending
NCT04025216	The sponsor finds the risk/benefit analysis unfavorable and has terminated the study.	A Phase 1 Open-Label, Multi-Center First in Human Study of TnMUC1-Targeted Genetically-Modified Chimeric Antigen Receptor T Cells in Patients With Advanced TnMUC1-Positive Solid Tumors and Multiple Myeloma	Biological: CART-TnMUC1 Drug: Cyclophosphamide Drug: Fludarabine	Interventional, Phase 1, Parallel arms with sequential dose escalation, open label, ~16 participants	Kite, A Gilead Company, United States	As the study was terminated before completion, no results were posted. The termination was based on an unfavorable risk/benefit analysis by the sponsor.
NCT05483491	The trial is actively recruiting participants.	T Cell Receptor Gene Therapy Targeting KK-LC-1 for Gastric, Breast, Cervical, Lung, and Other KK-LC-1 Positive Cancers	Biological: KK-LC-1 TCR-T cells Drug: Aldesleukin	Interventional, Phase 1, Sequential Assignment, open label, ~30 participants	The State University of New Jersey, United States	Results pending
NCT04981119	The trial is actively recruiting participants.	An Observational Study Obtaining Solid Tumor Tissue From Participants and Apheresis for CAR T-Cell Therapy Manufacturing	Other: Apheresis Diagnostic Test: Next Generation Sequencing (NGS) Diagnostic Test: Long Range NGS HLA typing	Observational, ~200 participants	A2 Biotherapeutics Inc., United States	Results pending
NCT05694364	The trial is active but not recruiting participants.	A Phase 1/1b Dose Escalation/Dose Expansion Study of PRGN-3007 UltraCAR-T Cells in Patients With Advanced Hematologic and Solid Tumor Malignancies	Drug: Fludarabine Drug: Cyclophosphamide Biological: PRGN-3007	Interventional, Phase 1/1b, Dose Escalation/Dose Expansion, open label, 3 participants (actual)	H. Lee Moffitt Cancer Center and Research Institute, United States	Results pending
NCT05035407	The trial is actively recruiting participants.	A Phase I Trial of T Cell Receptor Gene Therapy Targeting KK-LC-1 for Gastric, Breast, Cervical, Lung and Other KK-LC-1 Positive Epithelial Cancers	Drug: IL-2 (Aldesleukin) Drug: Cyclophosphamide Biological: KK-LC-1 TCR Drug: Fludarabine	Interventional, Phase 1, Sequential Assignment, open label, ~30 participants	National Institutes of Health Clinical Center (CC) (National Cancer Institute (NCI)), Unites States	Results pending

**Table 3:** Completed and ongoing CAR T-cell therapy clinical trials recruiting lung cancer patients. Table as of 19 August 2025.

NCT Number	Study Status	Study Title	Interventions	Design	Sponsor Country and Institution	Key Results
NCT06972576	Recruiting	Clinical Study of Combined EphA2-targeted CAR-DC and CAR-T Cell Therapy for Non-small Cell Lung Cancer	Biological: EphA2-targeted CAR-T Cells Biological: EphA2-targeted CAR-DCs	Interventional, Phase 1, open label, ~18 participants	Second Affiliated Hospital, School of Medicine, Zhejiang University, China	Results pending
NCT05060796	Recruiting	A Single-arm, Open-label, Phase I Study to Evaluate the Safety and Efficacy of CXCR5 Modified EGFR Chimeric Antigen Receptor Autologous T Cells in EGFR-positive Patients With Advanced Non-small Cell Lung Cancer	Biological: CXCR5 modified EGFR Chimeric Antigen Receptor Autologous T cells	Interventional, Phase 3, Parallel Assignment, open Label, ~11 participants	Second Affiliated Hospital of Guangzhou Medical University, China	Results pending
NCT06043466	Recruiting	Phase I Clinical Study of Chimeric Antigen Receptor T Cells (C-13-60) in the Treatment of Carcinoembryonic Antigen (CEA) Positive Advanced Malignant Solid Tumors	Biological: CEA-targeted CAR-T cells	Interventional, Phase 1, Sequential Assignment, open Label, ~30 participants	Chongqing Precision Biotech Co., Ltd., China	Results pending
NCT06653023	Recruiting	A Clinical Study on the Safety and Efficacy of Universal CAR-T Cells (REVO-UWD-03) for Advanced Hepatocellular Carcinoma &Amp; Lung Cancer	Biological: Universal CAR-T cells injection for treating HCC and NSCLC	Interventional, Early Phase 1, open Label, ~60 participants	Wondercel Biotech (ShenZhen), China	Results pending
NCT06682793	Recruiting	A Seamless Phase 1/2 Study to Evaluate the Safety and Efficacy of A2B395, an Allogeneic Logic-gated Tmod™ CAR T, in Heterozygous HLA-A*02 Adults With Recurrent Unresectable, Locally Advanced, or Metastatic Solid Tumors That Express EGFR and Have Lost HLA-A*02 Expression	Biological: A2B395 Diagnostic Test: xT CDx with HLA-LOH assay	Interventional, Phase 1 and 2, open Label, ~240 participants	A2 Biotherapeutics Inc., United States	The key result so far is that: The first patient has been successfully dosed with A2B395 Tmod™ CAR T cells on 26 June 2025, marking the inaugural human administration of this innovative therapy
NCT06051695	Recruiting	A Seamless Phase 1/2 Study to Evaluate the Safety and Efficacy of A2B694, an Autologous Logic-gated Tmod™ CAR T, in Heterozygous HLA-A*02 Adults With Recurrent Unresectable, Locally Advanced, or Metastatic Solid Tumors That Express MSLN and Have Lost HLA-A*02 Expression	Biological: A2B694 Diagnostic Test: xT CDx with HLA-LOH Assay	Interventional, Phase 1 and 2, open Label, ~230 participants	A2 Biotherapeutics Inc., United States	First Patient Dosed: The trial successfully administered A2B694 to its first patient in April 2024, marking a major milestone in clinical translation. Dose Escalation: The dose-escalation phase is currently ongoing; no dosage outcomes or adverse event data have been published yet.
NCT05620342	Recruiting	Administration of T Cells Expressing a 2nd Generation GD2 Chimeric Antigen Receptor, IL-15, and iCaspase9 Safety Switch in Subjects With Lung Cancer	Biological: iC9.GD2.CAR.IL-15 T Infusion	Interventional, Early Phase 1, open Label, ~24 participants	UNC Lineberger Comprehensive Cancer Center, United States	Results pending
NCT02587689	Last known status was: Recruiting	Phase I/II Study of Anti-MUC1 CAR T Cells for Patients With MUC1+ Advanced Refractory Solid Tumor	Biological: anti-MUC1 CAR T-Cells	Interventional, Phase 1 and 2, open Label, ~20 participants	PersonGen BioTherapeutics (Suzhou) Co., Ltd., China	Results pending
NCT04503278	Recruiting	Phase I/IIa, First-in-human (FIH), Open-label, Dose Escalation Trial With Expansion Cohorts to Evaluate Safety and Preliminary Efficacy of CLDN6 CAR-T With or Without CLDN6 RNA-LPX in Patients With CLDN6-positive Relapsed or Refractory Advanced Solid Tumors	Biological: CLDN6 CAR-T Biological: CLDN6 uRNA-LPX/CLDN6 modRNA-LPX	Interventional, Phase 1, open Label, ~214 participants	BioNTech Cell & Gene Therapies GmbH, Germany Study Locations: Australia, Germany, Netherlands, Sweden	Results pending
NCT04981119	Recruiting	An Observational Study Obtaining Solid Tumor Tissue From Participants and Apheresis for CAR T-Cell Therapy Manufacturing	Other: Apheresis Diagnostic Test: Next Generation Sequencing (NGS) Diagnostic Test: Long Range NGS HLA typing	Observational, ~200 participants	A2 Biotherapeutics Inc., United States	About 4–5% had tumors with HLA-A02 LOH. Over 30 patients have successfully undergone leukapheresis to bank T-cells for future Tmod CAR-T trials (EVEREST studies). Recruitment efficiency improved with digital screening tools (AWARE system).

**Table 4:** Completed and ongoing CAR T-cell therapy clinical trials recruiting renal cell carcinoma patients. Table as of 19 August 2025.

NCT Number	Study Status	Study Title	Interventions	Design	Sponsor Country and Institution	Key Results
NCT06586658	Recruiting	An Exploratory Clinical Study of the Safety and Efficacy of Anti-CD70-CAR-T Cell Injection in Patients With Locally Advanced or Relapsed/Metastatic Renal Cell Carcinoma With CD70+ Inoperable	Biological: anti-CD70-CAR-T cells	Interventional, Early Phase 1, open Label, ~9 participants	Shanghai Changzheng Hospital, China	Results pending
NCT05420519	Last known status was: Recruiting	A Phase I Clinical Study of CD70-targeted CAR-T Therapy for Advanced/Advanced Renal Cancer	Biological: CD70 CAR-T cells	Interventional, Phase 1, open Label, ~24 participants	Chongqing Precision Biotech Co., Ltd., China	Results pending
NCT04969354	Recruiting	Clinical Study of CAIX-targeted CAR-T Cells in the Treatment of Advanced Renal Cell Carcinoma	Biological: CAR-T cell immunotherapy	Interventional, Phase 1, open Label, ~20 participants	The Affiliated Hospital of Xuzhou Medical University, China	Results pending
NCT06010875	Recruiting	A Phase I Clinical Study to Assess the Safety and Efficacy of CD70-targeted CAR-T in the Treatment of CD70-positive Advanced/Metastatic Solid Tumors	Biological: CD70 CAR-T cells Biological: CD70 CAR-T cells	Interventional, Phase 1, open Label, ~48 participants	Chongqing Precision Biotech Co., Ltd., China	Results pending
NCT05518253	Recruiting	A Phase I Clinical Study of CD70-targeting CAR-T Therapy in the Treatment of CD70-positive Advanced/Metastatic Solid Tumors	Biological: CD70 CAR-T cells Biological: CD70 CAR-T cells	Interventional, Phase 1, open Label, ~30 participants	Zhejiang University, China	Results pending
NCT05420545	Last known status was: Recruiting	A Phase I Clinical Study of CD70-targeting CAR-T Therapy in the Treatment of CD70-positive Advanced/Metastatic Solid Tumors	Biological: CD70 CAR-T cells Biological: CD70 CAR-T cells	Interventional, Phase 1, open Label, ~36 participants	Chongqing Precision Biotech Co., Ltd., China	Results pending
NCT05468190	Last known status was: Recruiting	A Phase I Clinical Study to Assess the Safety and Tolerability of CD70-targeting CAR-T in the Treatment of CD70-positive Advanced/Metastatic Solid Tumors	Biological: CD70 CAR-T cells Biological: CD70 CAR-T cells	Interventional, Phase 1, open Label, ~48 participants	Chongqing Precision Biotech Co., Ltd., China	Results pending
NCT03393936	Terminated, Adjustment of study strategy	A Dose Escalation and Dose Expansion Trial to Assess the Safety, Tolerability and Anti-tumor Activity of Autologous T Cell Modified Chimeric Antigen Receptor (CAR) CCT 301-38 or CCT 301-59 in Patients With Recurrent or Refractory Stage IV Renal Cell Carcinoma	Biological: CCT301-38 Biological: CCT301-59	Interventional, Phase 1 and 2, open Label, ~66 participants	Shanghai PerHum Therapeutics Co., Ltd., China	Results pending
NCT06480565	Active, not recruiting	A Phase 1/2 Trial of ADI-270 (Engineered γδ Chimeric Receptor [CAR] Vδ1 T Cells Targeting CD70) in Adults With Relapsed or Refractory (R/R) Clear Cell Renal Cell Carcinoma (ccRCC)	Drug: ADI-270 Drug: Fludarabine Drug: Cyclophosphamide	Interventional, Phase 1 and 2, open Label, ~60 participants	Adicet Therapeutics, United States	Results pending
NCT04438083	Terminated, Patients to be followed up in the CRSP-ONC-LTF study	A Phase 1 Dose Escalation and Cohort Expansion Study of the Safety and Efficacy of Allogeneic CRISPR-Cas9-Engineered T Cells (CTX130) in Subjects With Advanced, Relapsed or Refractory Renal Cell Carcinoma With Clear Cell Differentiation	Biological: CTX130	Interventional, Phase 1, open Label, ~19 participants	CRISPR Therapeutics AG, Study Locations: United States, Australia, Canada, Netherlands	Results pending
NCT01218867	Terminated, No objective responses were observed	Phase I/II Study of Metastatic Cancer Using Lymphodepleting Conditioning Followed by Infusion of Anti-VEGFR2 Gene Engineered CD8+ Lymphocytes	Biological: Anti-VEGFR2 CAR CD8 plus PBL Drug: Cyclophosphamide Biological: Aldesleukin Drug: Fludarabine	Interventional, Phase 1 and 2, open Label, ~24 participants	National Cancer Institute (NCI), United States	Terminated Phase I/II; showed early signs of antitumor activity—9 responses, with up to 12 patients achieving their best response within one month post-infusion.
NCT04696731	Recruiting	A Phase 1 Multicenter Study Evaluating the Safety and Efficacy of ALLO-316 Following ALLO-647 Containing Conditioning Regimen in Subjects With Advanced or Metastatic Clear Cell Renal Cell Carcinoma	Genetic: ALLO-316 Biological: ALLO-647 Drug: Fludarabine Drug: Cyclophosphamide	Interventional, Phase 1, open Label, ~120 participants	Allogene Therapeutics, United States	Results pending
NCT05795595	Recruiting	A Phase 1/2, Open-label, Multicenter, Dose Escalation and Cohort Expansion Study of the Safety and Efficacy of Anti-CD70 Allogeneic CRISPR-Cas9-Engineered T Cells (CTX131) in Adult Subjects With Relapsed or Refractory Solid Tumors	Biological: CTX131	Interventional, Phase 1 and 2, open Label, ~250 participants	CRISPR Therapeutics AG, United States	Results pending
NCT02830724	Recruiting	A Phase I/II Study Administering Peripheral Blood Lymphocytes Transduced With a CD70-Binding Chimeric Antigen Receptor to Patients With CD70-Expressing Cancers	Drug: Cyclophosphamide Drug: Fludarabine Drug: Aldesleukin Biological: Anti-hCD70 CAR transduced PBL	Interventional, Phase 1 and 2, open Label, ~124 participants	National Cancer Institute (NCI), United States	Results pending
